# Cardiac electrical remodeling in Graves’ disease: insights from global ventricular repolarization indices

**DOI:** 10.3389/fendo.2026.1979644

**Published:** 2026-09-16

**Authors:** Maksymilian Kłosowicz, Magdalena Urbańczuk, Aleksandra Burbelka, Agnieszka Gala-Błądzińska, Krzysztof Balawender

**Affiliations:** 1 Department of Normal and Clinical Anatomy, Faculty of Medicine, Medical College, University of Rzeszów, Rzeszów, Poland; 2 Clinical Department of Cardiology with the Acute Coronary Syndromes Subdivision, St. Queen Jadwiga Clinical Hospital in Rzeszow, Rzeszow, Poland; 3 Department of Internal Medicine, Nephrology, and Endocrinology, St. Queen Jadwiga Clinical Hospital in Rzeszow, Rzeszow, Poland; 4 Department of Nephrology and Endocrinology, Faculty of Medicine, Medical College, University of Rzeszów, Rzeszów, Poland; 5 Department of Nephrology and Dialysis Unit, Fryderyk Chopin University Clinical Hospital in Rzeszów, Rzeszów, Poland

**Keywords:** electrocadiography, global repolarization parameters, Graves disease (GD), JT interval, overt hyperthyroidism, Tp-e interval, Tp-e/QT

## Abstract

**Background:**

Thyroid hormone excess can affect cardiac electrical activity through several mechanisms, yet its relationship with ventricular repolarization remains incompletely understood. Standard electrocardiographic intervals primarily characterize the overall duration of repolarization, whereas newer ECG-derived indices may provide additional information on its spatial and temporal heterogeneity. A better understanding of these alterations may be clinically relevant in light of emerging evidence linking circulating thyroid hormone levels with sudden cardiac death.

**Objectives:**

The aim of this study was to identify electrocardiographic parameters of ventricular repolarization that exhibit the most pronounced alterations in patients with Graves’ disease, thereby characterizing the pattern of repolarization changes associated with thyroid hormone excess.

**Methods:**

We conducted an observational analysis comparing individuals with patients with Graves’ disease with age- and sex-matched euthyroid controls. Ventricular repolarization was assessed using QT, QTc, JT, JTc, Tp-e, and Tp-e–based ratios. Group differences and discriminatory performance were evaluated using non-parametric tests and ROC analysis. Independent associations with hyperthyroidism were examined using multivariable logistic regression with entropy balancing and internal bootstrap validation.

**Results:**

Compared with controls, patients with Graves’ disease showed higher Tp-e values and Tp-e–derived ratios, consistent with greater heterogeneity of ventricular repolarization. Conversely, the raw QT and JT intervals were reduced, while their rate-corrected counterparts (QTc and JTc) showed only limited differences that varied according to the correction method. Among the evaluated ECG measures, Tp-e/QT provided the strongest separation between the groups, yielding an AUC of 0.92 (95% CI 0.84–0.97), with a sensitivity of 83% and specificity of 86% at a threshold of ≥0.24. After multivariable adjustment, Tp-e/QT remained independently related to overt hyperthyroidism (OR 1.75 for each 0.01 increment, 95% CI 1.31–2.34; p < 0.001). Heart rate was also independently associated with hyperthyroidism (OR 1.05 per 1-bpm increase, 95% CI 1.00–1.10; p = 0.046).

**Conclusions:**

In patients with Graves’ hyperthyroidism, ventricular repolarization appears to be characterized by greater electrical heterogeneity without a consistent increase in overall repolarization duration. Tp-e–derived measures may capture these abnormalities more effectively and provide additional information beyond standard ECG repolarization indices.

## 1 Introduction

Overt hyperthyroidism (OHT) is a clinical condition characterized by suppressed levels of thyroid-stimulating hormone (TSH) with concomitantly elevated concentrations of free thyroid hormones – free triiodothyronine (FT3) and/or free thyroxine (FT4). The prevalence of OHT in the general population is estimated at approximately 0.2–1.4%. Among its various etiologies, Graves’ disease is the predominant cause and represents the most common form of endogenous hyperthyroidism. Less frequently, hyperthyroidism arises from toxic multinodular goiter and toxic adenoma, thyroiditis or iatrogenic factors such as inappropriate L-thyroxine therapy, amiodarone-induced thyrotoxicosis, or exposure to iodine-containing contrast agents (1).

The cardiovascular system is particularly sensitive to changes in thyroid hormone (TH) activity due to the widespread expression of TH receptors within the myocardium and vascular tissues. In hyperthyroidism, excess circulating thyroid hormones produce a hyperdynamic cardiovascular state characterized by increased heart rate and myocardial contractility, together with alterations in vascular tone and endothelial function. Thyroid hormone excess also affects lipid metabolism and may promote atherosclerotic processes. These cardiovascular effects contribute to the clinical manifestations of hyperthyroidism and are associated with an increased occurrence of heart failure and cardiac arrhythmias, most notably atrial fibrillation (AF) (2–4).

Electrocardiography (ECG) is a widely available, non-invasive, and inexpensive method for detecting cardiac electrical abnormalities. In hyperthyroidism, changes in conventional ECG measures, including QT and QTc intervals, have been well documented. More recently, attention has shifted toward additional ventricular repolarization indices, such as Tp-e, JT, JTc, and Tp-e–derived ratios, which can be obtained from standard 12-lead ECG recordings. These parameters may provide complementary information on ventricular repolarization and its heterogeneity beyond routinely assessed ECG measures (5).

In the present study, we focus on advanced markers of ventricular repolarization and repolarization heterogeneity – Tp-e, QT, QTc, QTd, Tp-e/QT, Tp-e/QTc, ST-segment abnormalities, and T-wave abnormalities. Alterations in these parameters reflect changes in ventricular repolarization and repolarization heterogeneity, some of which have been associated with arrhythmic outcomes in previous studies (5).

## 2 Materials and methods

### 2.1 Study population

The study cohort comprised patients with overt hyperthyroidism and euthyroid controls with comparable demographic characteristics. Based on the inclusion criteria, a total of 953 records were identified as eligible. After applying the exclusion criteria, 54 patients were ultimately qualified for further analysis. The detailed process of case selection is illustrated in **Figure 1**.

**Figure 1:**
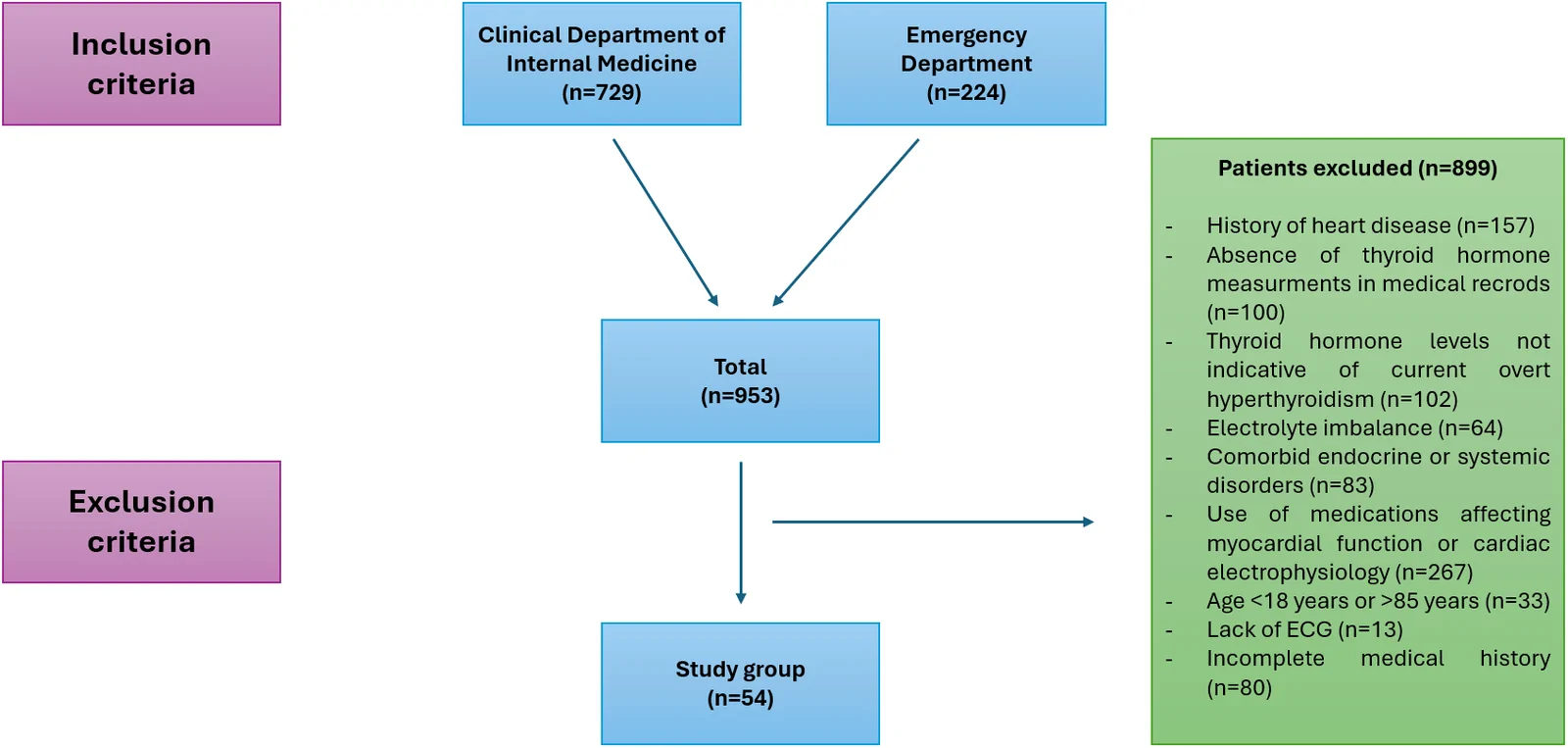
Process of case selection.

The study cohort included patients with Graves’ disease and biochemically confirmed overt hyperthyroidism. At the time of ECG acquisition, none of the included patients were receiving antithyroid therapy; therefore, electrocardiographic measurements reflected the untreated hyperthyroid state. For clarity, patients with Graves’ disease and overt hyperthyroidism are hereafter referred to as the OHT group.

To minimize the influence of factors known to affect ventricular depolarization and repolarization, patients receiving β-blockers or other medications affecting cardiac conduction or repolarization were excluded. Furthermore, individuals with significant cardiovascular diseases, including coronary artery disease, previous myocardial infarction, heart failure, cardiomyopathies, moderate or severe valvular heart disease, atrial fibrillation or other clinically significant arrhythmias, bundle branch block, congenital heart disease, cardiac implantable electronic devices, and inherited channelopathies, were also excluded. Additional exclusion criteria comprised electrolyte abnormalities and other conditions known to influence electrocardiographic parameters. All of the inclusion and exclusion criteria for patient enrolment are presented in detail in **Table 1**.

**Table 1:** Inclusion and exclusion criteria for patient enrolment.

Inclusion criteria	Exclusion criteria
Patients over 18 years of age	Previous history of heart disease
Hospitalization between 01.06.2022 – 30.06.2025	Current use of pharmacological agents exerting a direct influence on myocardial function or cardiac electrophysiology (e.g. β-blockers, antiarrhythmic drugs, digoxin, non-dihydropyridine calcium channel)
Hyperthyroidism diagnosed in the medical history	Lack of thyroid hormone measurements in medical records
	Electrolyte imbalances
	Comorbid endocrine or systemic disorders resulting in hormonal alterations capable of modifying ECG characteristics
	Thyroid hormone levels not indicative of current overt hyperthyroidism

### 2.2 Data collection

Data were collected at a tertiary care hospital in southeastern Poland. The study was conducted in accordance with the Declaration of Helsinki and approved by the local institutional ethics committee (approval no. KB/23/D/2025).

Biometric data and laboratory test results of selected patients were retrieved from the hospital’s electronic system. All extracted variables were subsequently entered into a dedicated, pre-designed Microsoft Excel spreadsheet developed specifically for this study, where the data were organized, coded, and prepared for further statistical analysis. All data were anonymized, with patient names replaced by identification numbers. The extracted data were supplemented with information from paper medical records and available electrocardiographic (ECG) tracings. In cases where multiple ECG recordings were available, the tracing performed closest to the date of blood sampling for thyroid hormone assays was selected for analysis.

### 2.3 ECG analysis

We used a standard 12-lead ECG tracing at 25-mm/s paper speed and 10-mm/mV amplitude. All electrocardiographic recordings were obtained with patients in the resting supine position. Electrocardiographic intervals were measured digitally using ImageJ software (National Institutes of Health, Bethesda, MD, USA). Calibration was performed according to the standard ECG grid (25 mm/s, 10 mm/mV), with horizontal scaling verified using a 1-second interval (five large squares = 1000 ms) and vertical scaling based on 1 mV (10 mm).

A standardized measurement protocol was established before the initiation of ECG analysis, with predefined criteria for lead selection, identification of waveform boundaries, and interval measurement. The protocol was consistently applied throughout the study to minimize measurement variability and promote consistency across measurements. To assess interobserver reproducibility, ECG recordings from a randomly selected subset of 50 participants, including 25 patients with OHT and 25 euthyroid controls, were independently re-evaluated by a second investigator blinded to the measurements obtained by the primary observer. Agreement between observers was assessed using the intraclass correlation coefficient (ICC) with 95% confidence intervals.

A detailed description of the measurement methodology, including the predefined criteria used for each ECG parameter, is provided in the **Supplementary Materials**. A graphical representation of the analyzed electrocardiographic parameters is presented in **Figure 2**.

**Figure 2:**
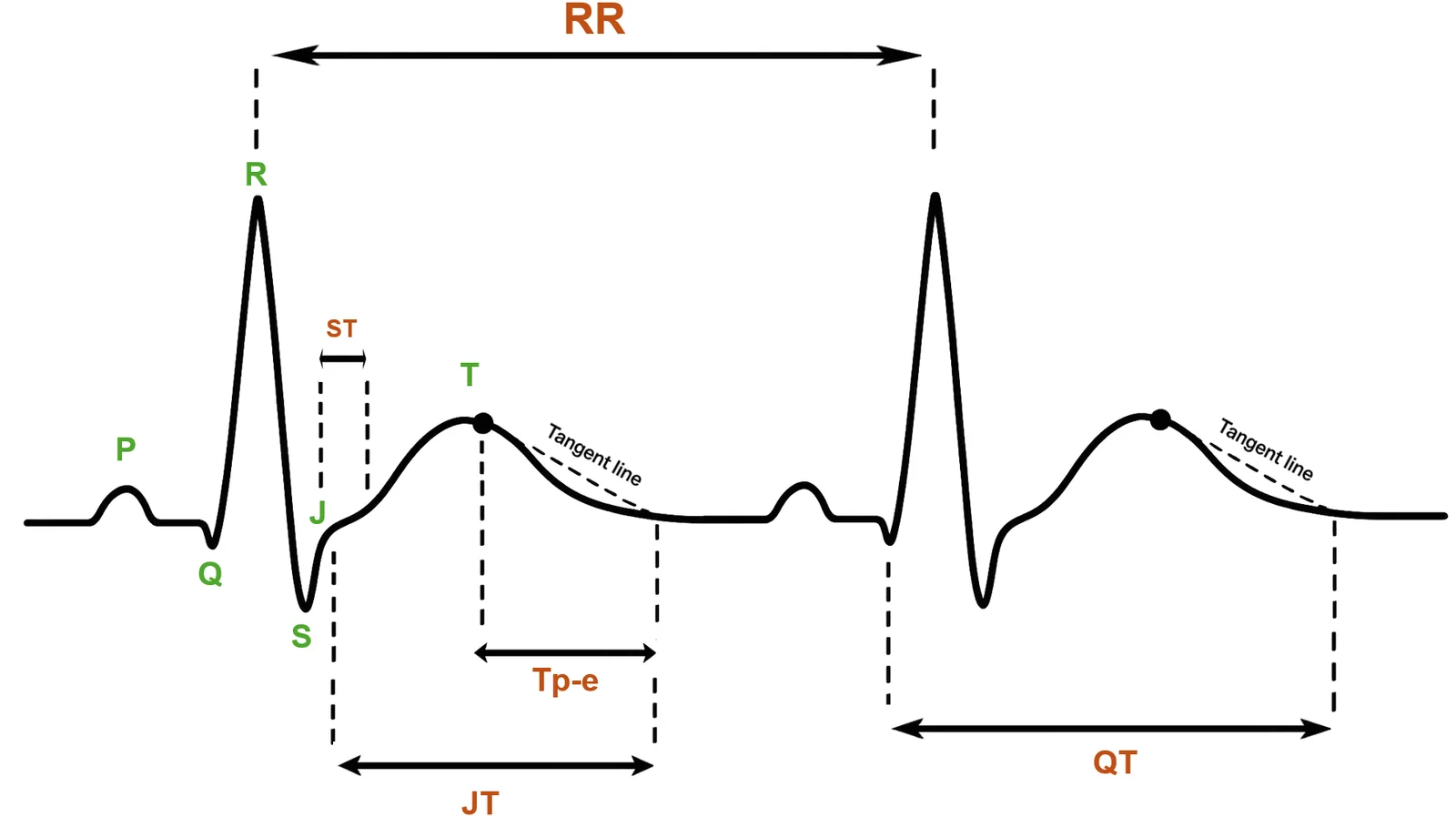
Graphical representation of the electrocardiographic intervals used for the assessment of ventricular repolarization. RR denotes the interval between consecutive R waves; QT, the interval from the onset of the QRS complex to the end of the T wave; JT, the interval from the J point to the end of the T wave; and Tp-e, the interval from the peak to the end of the T wave. The end of the T wave was determined using the tangent method.

All ECG measurements were accurately attributed to individual patients and recorded in the purpose-built Excel spreadsheet used for standardized data organization and analysis.

### 2.4 Statistical analysis

#### 2.4.1 Statistical significance, descriptive statistics and tests

Statistical analyses were conducted with a predefined significance level (α) of 0.05 to determine statistical significance across all tests. Descriptive statistics for continuous variables included medians accompanied by interquartile ranges (first quartile to third quartile), 95% confidence intervals calculated using the Wilcoxon method for medians, and ranges encompassing minimum and maximum values. For categorical variables, frequencies were reported as counts with percentages, supplemented by 95% confidence intervals derived via the Wilson score method.

Group comparisons of continuous variables employed the Wilcoxon rank-sum test, while categorical variables were assessed using Pearson’s chi-squared test.

#### 2.4.2 Correlation analysis

Associations between electrocardiographic parameters and thyroid function markers within the overt hyperthyroidism subgroup were assessed using Spearman rank correlation coefficients. Given the exploratory nature of this analysis, aimed at identifying preliminary patterns rather than confirming hypotheses, no adjustments for multiple comparisons were applied.

#### 2.4.3 Optimal cutpoint analysis

The discriminatory performance of electrocardiographic parameters in distinguishing patients with overt hyperthyroidism from healthy controls was evaluated using receiver operating characteristic (ROC) analysis, with optimal cut-off values determined by maximizing Youden’s index (sensitivity + specificity - 1) to balance true positive and true negative rates (Youden, 1950). Performance metrics included accuracy, sensitivity, specificity, true positives (TP), false negatives (FN), false positives (FP), and true negatives (TN), with the positive class defined as the presence of overt hyperthyroidism. The area under the ROC curve (AUC) served as the primary measure of discriminatory ability, with 95% confidence intervals estimated via 1000 bootstrap resamples to account for sampling variability and provide robust interval estimates.

#### 2.4.4 Propensity score weighting procedure

To address potential confounding and achieve covariate balance between the hyperthyroidism and control groups, entropy balancing was implemented as a propensity score weighting procedure, enabling estimation of the average treatment effect in the population. Detailed information regarding optimization constraints, balance diagnostics, weight stability metrics, and effective sample size assessment is provided in the **Supplementary Material**.

#### 2.4.5 Multivariable logistic regression analysis

Extended methodological details related to penalized regression procedures, model diagnostics, and internal validation—including least absolute shrinkage and selection operator (LASSO) regularization paths, cross-validation profiles, and bootstrap-derived performance metrics - are provided in the **Supplementary Material**.

## 3 Results

### 3.1 Demographic, clinical, and electrocardiographic parameters stratified by study group

The study cohort comprised patients with Graves’ disease (N = 54) and healthy controls (N = 42), with demographic, clinical, and electrocardiographic profiles compared to elucidate group differences, as summarized in **Table 2**.

**Table 2:** Comparison of demographic, clinical, and electrocardiographic parameters between patients with hyperthyroidism and healthy controls.

Characteristic	OHT (N = 54)	Control (N = 42)	p-value
I. Demographic characteristics			
Age (years)	45.50 (35.00-59.00), CI: 44, 52;	46.50 (38.00-59.00), CI: 44, 54; [20.00, 85.00]	0.690
Sex:			0.224
Female	42 (77.78%), CI: 64, 88	28 (66.67%), CI: 50, 80	
Male	12 (22.22%), CI: 12, 36	14 (33.33%), CI: 20, 50	
BMI (kg/m²)	21.64 (20.52-24.50), CI: 21, 23; [17.00, 29.27]	22.75 (21.20-23.70), CI: 22, 23; [18.50, 26.80]	0.639
II. Clinical parameters			
TSH (uIU/ml)	0.01 (0.01-0.01), CI: 0.01, 0.01; [<0.001, 0.53]	1.74 (1.47-2.41), CI: 1.70, 2.20; [0.69, 3.90]	< 0.001
HR (beats/min)	102.50 (93.00-115.00), CI: 97, 108; [65.00, 152.00]	74.44 (65.79-86.46), CI: 70, 79; [47.06, 102.21]	< 0.001
III. Electrocardiographic parameters			
QT interval in the same lead as Tp-e (ms)	355.00 (328.00-373.00), CI: 344, 361; [293.00, 454.00]	382.50 (363.00-402.00), CI: 374, 395; [307.00, 463.00]	< 0.001
QTc (Bazett) in the same lead as Tp-e (ms)	457.34 (442.49-475.98), CI: 450, 466; [366.37, 540.98]	424.85 (412.12-441.34), CI: 418, 434; [362.98, 479.57]	< 0.001
QTc (Fridericia) in the same lead as Tp-e (ms)	417.95 (405.30-436.07), CI: 413, 427; [343.14, 470.75]	410.47 (398.26-426.13), CI: 404, 419; [351.90, 463.87]	0.090
JT interval in the same lead as Tp-e (ms)	263.00 (246.00-280.00), CI: 256, 271; [212.00, 373.00]	293.50 (271.00-316.00), CI: 282, 304; [226.00, 365.00]	< 0.001
JTc (Bazett) in the same lead as Tp-e (ms)	0.34 (0.32-0.36), CI: 0.33, 0.35; [0.30, 0.42]	0.32 (0.31-0.34), CI: 0.32, 0.33; [0.26, 0.38]	0.003
JTc (Fridericia) in the same lead as Tp-e (ms)	0.32 (0.29-0.33), CI: 0.31, 0.32; [0.27, 0.39]	0.31 (0.30-0.33), CI: 0.31, 0.32; [0.26, 0.37]	1.000
Tp-e interval (ms)	101.00 (90.00-110.00), CI: 96, 104; [74.00, 140.00]	83.00 (76.00-89.00), CI: 80, 87; [64.00, 111.00]	< 0.001
Tp-e/QT ratio	0.28 (0.25-0.32), CI: 0.27, 0.30; [0.21, 0.39]	0.21 (0.20-0.24), CI: 0.21, 0.23; [0.17, 0.27]	< 0.001
Tp-e/QTc (Fridericia) ratio	0.24 (0.22-0.26), CI: 0.23, 0.25; [0.18, 0.33]	0.20 (0.19-0.22), CI: 0.20, 0.21; [0.16, 0.26]	< 0.001

Data are presented as median (Q1-Q3), CI: lower limit, upper limit; for continuous variables and n (%), CI: lower limit, upper limit for categorical variables. Wilcoxon rank sum test for continuous variables; Pearson’s Chi-squared test for categorical variables. Confidence intervals are calculated using the Wilcoxon method for medians of continuous variables and the Wilson score method for proportions of categorical variables. BMI, body mass index; TSH, thyroid-stimulating hormone; HR, heart rate; QT, QT interval; QTc, corrected QT interval; JT, JT interval; JTcb, JT interval corrected by Bazett formula; JTcf, JT interval corrected by Fridericia formula; Tp-e, T-peak to T-end interval; ms, milliseconds; uIU/ml, microinternational units per milliliter; kg/m², kilograms per square meter; CI, confidence interval; Q1, first quartile; Q3, thirdquartile.

Demographically, the groups were matched, without pronounced confounding influences on electrocardiographic outcomes. Median ages were similar (OHT: 45.50 years; controls: 46.50 years; p = 0.690). Sex distribution favored females in both (OHT: 77.78%; controls: 66.67%; p = 0.224). Body mass index (BMI) showed no significant difference (OHT: 21.64 kg/m²; controls: 22.75 kg/m²; p = 0.639), with both medians falling within the normal range (18.5–24.9 kg/m²).

Marked differences were observed in thyroid function and resting heart rate. Thyroid-stimulating hormone (TSH) was markedly suppressed in OHT (median: 0.01 µIU/mL; range: <0.001–0.53), well below the normal reference range, confirming diagnostic suppression and distinguishing it from the euthyroid controls (median: 1.74 µIU/mL; p < 0.001), which resided comfortably within normal limits. Resting heart rate (HR) was significantly elevated in OHT (median: 102.50 bpm; range: 65–152), exceeding the normal adult range and indicative of sinus tachycardia. In contrast, controls exhibited a normal median HR of 74.44 bpm (p < 0.001).

Electrocardiographic profiles revealed significant differences in several ventricular repolarization parameters between patients with overt hyperthyroidism and healthy controls. The uncorrected QT interval was shortened in OHT (median: 355.00 ms) compared to controls (382.50 ms; p < 0.001). However, rate-corrected measures indicated prolongation: QTc (Bazett) in OHT reached 460.00 ms (upper normal/prolonged, exceeding typical thresholds of <440 ms for men and <460 ms for women), versus 410.00 ms in controls (normal; p < 0.001). Similarly, QTc (Fridericia) was 426.00 ms in OHT (approaching upper limits, often <440 ms) compared to 412.00 ms in controls (p = 0.001).

The JT interval, representing ventricular repolarization excluding depolarization, was reduced in OHT (median: 256.00 ms) relative to controls (300.00 ms; p < 0.001). Corrected JTc (Bazett) was mildly elevated at 359.00 ms in OHT versus 340.00 ms in controls (p < 0.001), while JTc (Fridericia) showed no difference (333.00 ms vs. 336.00 ms; p = 0.461). The T-peak to T-end (Tp-e) interval, a marker of transmural dispersion, was prolonged in OHT (96.00 ms) compared to controls (80.00 ms; p < 0.001).

Dispersion ratios further highlighted these between-group differences: Tp-e/QT was 0.27 in OHT (elevated beyond normal <0.25) versus 0.21 in controls (p < 0.001), and Tp-e/QTc (Fridericia) was 0.22 (slightly elevated) versus 0.19 (p < 0.001).

**Supplementary Table 1** provides additional characteristics within Biochemical and Hematological Parameters among patients with Grave’s disease. Their thyroid function was profoundly deranged, with median free triiodothyronine at 8.53 pg/mL (interquartile range 5.67–17.28) and free thyroxine at 3.25 ng/dL (interquartile range 2.43–4.71). Renal function showed mild hyperfiltration, evidenced by an elevated estimated glomerular filtration rate of 110 mL/min/1.73 m² (interquartile range 96–119) and reduced creatinine at 0.64 mg/dL (interquartile range 0.47–0.80). Among hematological parameters, there was subtle anemia, with median hemoglobin at 12.90 g/dL (interquartile range 12.05–14.20) and hematocrit at 38.10% (interquartile range 35.10–41.10). Electrolyte balance remained largely stable, with sodium at 140 mmol/L (interquartile range 138–141), potassium at 4.20 mmol/L (interquartile range 4.00–4.40), chloride at 106 mmol/L (interquartile range 103–108), and calcium at 9.40 mg/dL (interquartile range 9.21–9.76), indicating no significant disturbances.

### 3.2 Correlation analysis between electrocardiographic parameters and thyroid function markers in patients with Graves’ disease

Correlations between thyroid function markers (FT3, FT4, and TSH) and ventricular repolarization parameters were evaluated in patients with Graves’ hyperthyroidism and are summarized in **Figure 3**. Overall, most correlations were weak and did not reach statistical significance, indicating limited associations between the biochemical severity of thyroid dysfunction and the evaluated ECG indices.

**Figure 3:**
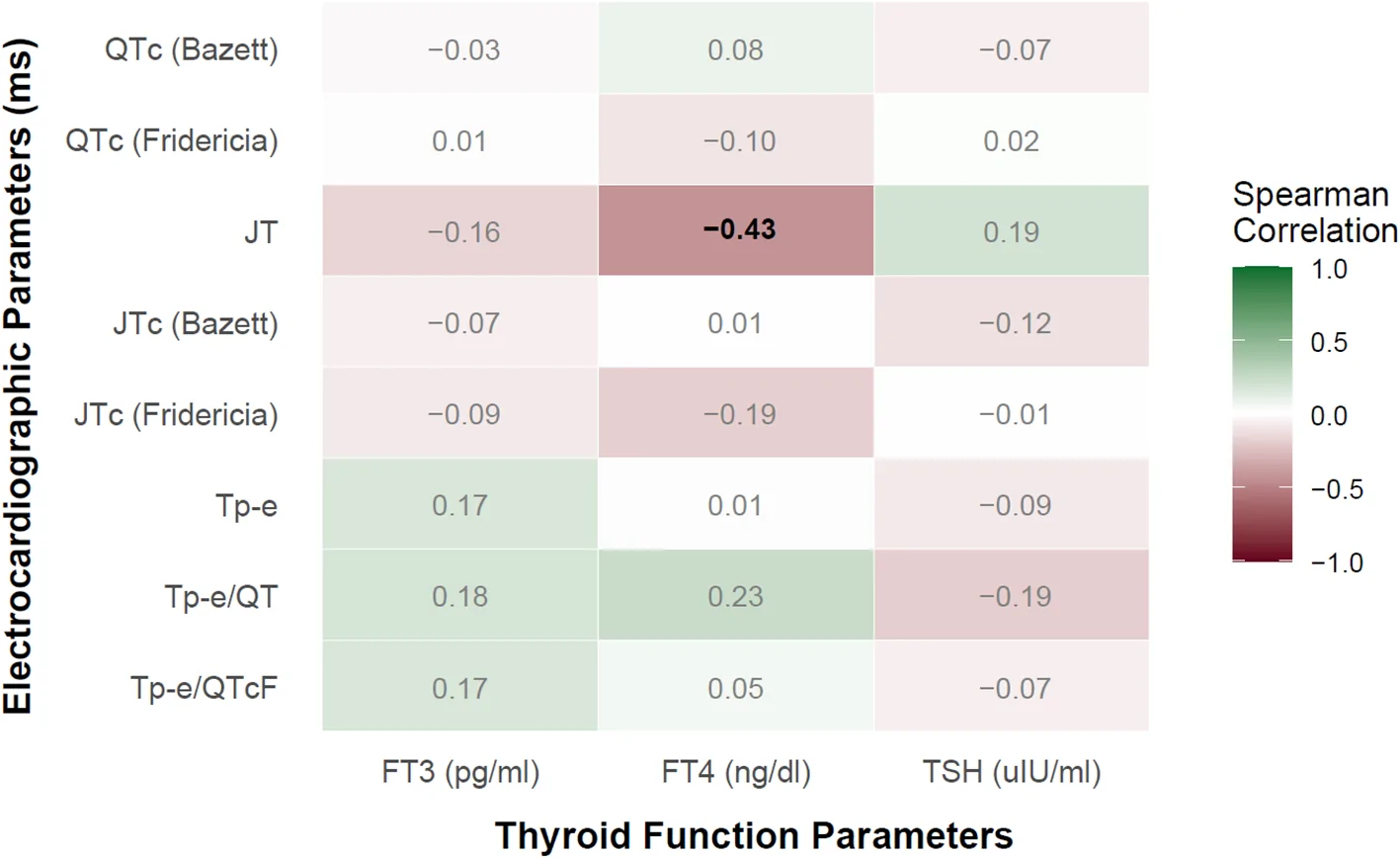
Spearman rank correlation heatmap depicting associations between electrocardiographic parameters and thyroid function markers (FT3, FT4, and TSH) in patients with OHT (N = 54).

The strongest association was observed between FT4 and the JT interval, with a moderate inverse correlation (ρ = −0.43, p = 0.001). A weaker positive relationship was found between FT4 and Tp-e/QT (ρ = 0.23, p = 0.089), although it did not reach statistical significance. Correlations of FT4 with QTcB (ρ = 0.08, p = 0.561), QTcF (ρ = −0.10, p = 0.465), JTcB (ρ = 0.01, p = 0.959), JTcF (ρ = −0.19, p = 0.180), Tp-e (ρ = 0.01, p = 0.935), and Tp-e/QTcF (ρ = 0.05, p = 0.708) were weak and non-significant.

FT3 was not significantly associated with any of the analyzed repolarization measures, with correlation coefficients ranging from ρ = −0.16 for JT (p = 0.236) to ρ = 0.18 for Tp-e/QT (p = 0.194). Similarly, correlations involving TSH were weak and non-significant, ranging from ρ = −0.19 for Tp-e/QT (p = 0.160) to ρ = 0.19 for JT (p = 0.170).

Thus, apart from the inverse association between FT4 and JT, no statistically significant relationships were identified between thyroid function markers and the evaluated ventricular repolarization indices within the Graves’ hyperthyroidism group.

### 3.3 Discriminatory performance of electrocardiographic parameters in Graves’ disease: receiver operating characteristic analysis

**Table 3** summarizes the ROC analysis evaluating the ability of eight electrocardiographic parameters to distinguish patients with Graves’ hyperthyroidism from healthy controls.

**Table 3:** Optimal cut-off values and discriminatory performance metrics for electrocardiographic parameters in distinguishing overt hyperthyroidism from healthy control.

Parameter	n	Optimal cut-off value	Youden index	Accuracy	Sensitivity	Specificity	TP	FN	FP	TN	AUC	95% CI
QTc (Bazett) in the same lead as Tp-e (ms)	96	≥442.5	0.55	0.77	0.76	0.79	41	13	9	33	0.80	0.67–0.92
QTc (Fridericia) in the same lead as Tp-e (ms)	96	≥415.5	0.21	0.60	0.59	0.62	32	33	16	26	0.60	0.45–0.77
JT interval in the same lead as Tp-e (ms)	96	≤268.0	0.43	0.71	0.65	0.79	35	19	9	33	0.75	0.61–0.87
JTc (Bazett) in the same lead as Tp-e (ms)	96	≥0.34	0.35	0.67	0.61	0.74	33	21	11	31	0.68	0.53–0.83
JTc (Fridericia) in the same lead as Tp-e (ms)	96	≥0.27	0.10	0.60	1.00	0.10	54	0	38	4	0.50	0.34–0.68
Tp-e interval (ms)	96	≥90.0	0.52	0.76	0.76	0.76	41	13	10	32	0.81	0.69–0.92
Tp-e/QT ratio	96	≥0.24	0.69	0.84	0.83	0.86	45	9	6	36	0.92	0.84–0.97
Tp-e/QTc (Fridericia) ratio	96	≥0.21	0.55	0.78	0.83	0.71	45	9	12	30	0.81	0.70–0.91

Optimal cut-off values were determined by maximizing Youden’s index (sensitivity + specificity - 1). Performance metrics (accuracy, sensitivity, specificity) are expressed as proportions (range: 0–1). TP = true positive; FN = false negative; FP = false positive; TN = true negative. The positive class is defined as the presence of overt hyperthyroidism. AUC = area under the receiver operating characteristic curve; 95% CI = 95% confidence interval, estimated using 1000 bootstrap resamples. n = number of observations with available data for each parameter; missing data were not imputed. All measurements were performed in the same electrocardiographic lead as the Tp-e interval (primarily V5). QTc, corrected QT interval; JT, JT interval; JTc, corrected JT interval; Tp-e, T-peak to T-end interval; ms, milliseconds.

Among the evaluated parameters, the Tp-e/QT ratio showed the highest discriminatory performance, with an AUC of 0.92 (95% CI: 0.84–0.97). At the optimal cut-off of ≥0.24, Tp-e/QT achieved an accuracy of 0.84, sensitivity of 0.83, and specificity of 0.86, corresponding to 45 true-positive and 9 false-negative classifications. Tp-e (AUC: 0.81; 95% CI: 0.69–0.92) and Tp-e/QTcF (AUC: 0.81; 95% CI: 0.70–0.91) also demonstrated good discriminatory performance, with sensitivity and specificity exceeding 0.70. QTcB yielded an AUC of 0.80 (95% CI: 0.67–0.92), with a sensitivity of 0.76 and specificity of 0.79.

Lower discriminatory performance was observed for JT (AUC: 0.75; 95% CI: 0.61–0.87), JTcB (AUC: 0.68; 95% CI: 0.53–0.83), and QTcF (AUC: 0.60; 95% CI: 0.45–0.77). JTcF showed no meaningful discrimination between the groups (AUC: 0.50; 95% CI: 0.34–0.68). Although JTcF achieved a sensitivity of 1.00 at the selected threshold, its specificity was only 0.10, resulting in limited overall accuracy (0.60). Overall, Tp-e–based indices, particularly Tp-e/QT, provided the strongest separation between patients with Graves’ hyperthyroidism and healthy controls.

### 3.4 Exploratory multivariable logistic regression analysis of electrocardiographic parameters associated with Graves’ disease

Predictor selection was conducted using penalized regression via least absolute shrinkage and selection operator (LASSO) methodology, which identified the corrected QT interval (Bazett formula) in the same lead as Tp-e (ms), the corrected JT interval (Bazett formula) in the same lead as Tp-e (ms), the Tp-e/QT ratio, and heart rate as candidate variables at the minimum cross-validation error. Given the limited sample size, LASSO was used as an exploratory variable-selection approach, and the resulting multivariable model was considered hypothesis-generating rather than confirmatory. A more conservative selection, based on the one-standard-error rule, yielded a reduced set comprising the corrected QT interval (Bazett formula) in the same lead as Tp-e (ms), the Tp-e/QT ratio, and heart rate (refer to **Supplementary Figures S2, S3** for coefficient trajectories and cross-validation profiles). Variance inflation factors for the initial set were below 3.31, signifying no substantial multicollinearity, while the conservative set demonstrated even lower values (all VIFs < 1.13), reinforcing overall model stability.

A sequential analysis of deviance was performed to evaluate the incremental contribution of each predictor. This revealed significant deviance reductions for the corrected QT interval (Bazett formula) in the same lead as Tp-e (ms) (26.45, p < 0.001), the Tp-e/QT ratio (48.49, p < 0.001), and heart rate (4.56, p = 0.033). In contrast, the corrected JT interval (Bazett formula) in the same lead as Tp-e (ms) contributed negligibly (0.62, p = 0.433).

To enhance interpretability, the predictors were centered at predefined reference points^1^: the corrected QT interval (Bazett formula) at 442.5 ms, the Tp-e/QT ratio at 0.24, and heart rate at its median value for the population (89 bpm).

As detailed in **Table 4**, the exploratory multivariable logistic regression model showed good apparent fit, with a likelihood ratio chi-square of 79.86 on 3 degrees of freedom (p < 0.001), a Nagelkerke R² of 0.76, and a concordance index of 0.96, indicating high discriminatory performance within the study cohort. The coefficients reflect positive associations: each 1 ms increase in the centered corrected QT interval (Bazett formula) elevates the log-odds of overt hyperthyroidism by 0.02 (OR = 1.02, 95% CI: 0.99–1.05); each 0.01 unit increase in the centered Tp-e/QT ratio does so by 0.56 (OR = 1.75, 95% CI: 1.31–2.34); and each 1 bpm increase in centered heart rate by 0.05 (OR = 1.05, 95% CI: 1.00–1.10). Statistical significance was attained for the Tp-e/QT ratio (p < 0.001) and heart rate (p = 0.046), with the corrected QT interval (Bazett formula) approaching significance (p = 0.147).

**Table 4:** Coefficients, odds ratios, and wald tests for the exploratory multivariable logistic regression model associated with overt hyperthyroidism.

Predictor	Coefficient	SE	OR (95% CI)	Wald Z Statistic	p-value
Intercept	0.19	0.38	1.21 (0.58–2.53)	0.51	0.610
Corrected QT interval (Bazett formula) in the same lead as Tp-e (ms)¹	0.02	0.01	1.02 (0.99–1.05)	1.45	0.147
Tp-e/QT ratio²	0.56	0.15	1.75 (1.31–2.34)	3.78	< 0.001
Heart rate (bpm)³	0.05	0.02	1.05 (1.00–1.10)	1.99	0.046

¹Centered at 442.5 ms; coefficient and odds ratio represent change per 1 ms increase. ²Centered at 0.24; preliminary coefficient and standard error divided by 100 for scaling; coefficient and odds ratio represent change per 0.01 unit increase. ³Centered at the median value 89.9 bpm; coefficient and odds ratio represent change per 1 bpm increase. Coefficients represent changes in log-odds. Odds ratios (OR) are exponentiated coefficients with 95% confidence intervals (CI) calculated as exp(coefficient ± 1.96 × standard error). Wald Z statistics test the null hypothesis that the coefficient is zero. p-values are two-tailed. ms, milliseconds; bpm, beats per minute; Tp-e, T-peak to T-end interval; QT, QT interval; SE, standard error; OR, odds ratio; CI, confidence interval.

Internal bootstrap validation based on 1,000 resamples (n = 96), as summarized in **Table 5**, was used to estimate optimism in model performance. Although the optimism-corrected indices indicated preserved discrimination and calibration within the study cohort, internal validation does not eliminate the possibility of overfitting, particularly given the limited sample size. Key indices include an optimism-corrected Somers’ Dxy of 0.90 (original training value: 0.92; optimism: 0.02), reflecting excellent discrimination post-adjustment, and an optimism-corrected Nagelkerke R² of 0.73 (original training value: 0.76; optimism: 0.03), indicating sustained explanatory power. Additional metrics encompass an optimism-corrected Brier score of 0.12 (original training value: 0.10; optimism: 0.02), underscoring low prediction error; a calibration slope of 0.89 (original training value: 1.00; optimism: 0.11) and a maximum absolute calibration error of 0.11 (original training value: 0.00; optimism: 0.11). The unreliability index (U) of 0.01 and discrimination index (D) of 0.46 yield a quality index (Q = D - U) of 0.45, providing additional information on internally validated model performance. Confidence intervals derived from the bootstrap distribution support the precision of these estimates. Calibration performance is visually corroborated in **Supplementary Figure S4**, where the bias-corrected curve closely tracks the ideal line, with a mean absolute error of 0.022.

**Table 5:** Bootstrap validation indices for the final multivariable logistic regression model.

Index	Original value	Training value	Test value	Optimism	Optimism-corrected value	95% CI
Somers’ Dxy	0.91	0.91	0.90	0.01	0.90	0.83–0.98
Nagelkerke R²	0.76	0.77	0.74	0.03	0.73	0.60–0.86
Calibration Intercept	0.00	0.00	0.01	-0.01	0.01	-0.91–0.94
Calibration Slope	1.00	1.00	0.89	0.11	0.89	0.39–1.35
Maximum Absolute Calibration Error	0.00	0.00	0.11	-0.11	0.11	-0.02–0.33
Discrimination Index (D)	0.82	0.84	0.79	0.05	0.77	0.53–0.99
Unreliability Index (U)	-0.02	-0.02	0.02	-0.04	0.02	-0.05–1.39
Quality Index (Q)	0.84	0.86	0.77	0.10	0.75	-0.44–1.00
Brier Score (B)	0.09	0.08	0.09	-0.01	0.10	0.06–0.15
Gini Coefficient (g)	4.39	6.61	4.21	2.41	1.98	-346.84–6.25
Proportion of Explained Variation (gp)	0.45	0.45	0.45	0.00	0.45	0.41–0.50

Validation based on 1000 bootstrap resamples (n = 96 observations). Optimism represents the average difference between training and test performance, with corrected values adjusting for potential overfitting. Confidence intervals are derived from the bootstrap distribution. All values rounded to two decimal places. Dxy, Somers’ rank correlation; R², Nagelkerke pseudo-R²; Q, D - U.

For illustrative purposes, the nomogram presented in **Supplementary Figure S5** provides a graphical representation of the exploratory model and the relative contribution of its predictors within the study cohort; it should not be interpreted as a validated clinical decision-making tool.

In conclusion, in the exploratory multivariable analysis, the Tp-e/QT ratio showed the strongest independent association with overt hyperthyroidism among the evaluated ECG parameters. Each 0.01-unit increase in the Tp-e/QT ratio was associated with 75% higher odds of belonging to the OHT group (OR = 1.75, 95% CI: 1.31–2.34), while heart rate was also independently associated with group status (OR = 1.05 per bpm, 95% CI: 1.00–1.10). The corrected QT interval (Bazett formula) provided **Supplementary Information**, although its association was not statistically significant. This parsimonious model provides an exploratory framework for assessing the discriminatory value of ECG parameters related to ventricular repolarization heterogeneity. Although internal validation supported the stability of the model within the study cohort, prospective external validation in larger, independent populations is essential to confirm the generalizability of these findings.

### 3.5 Measurement reproducibility

A randomly selected sample of 50 participants (25 with OHT and 25 euthyroid controls) underwent repeated ECG assessment under the same measurement conditions to evaluate intraobserver agreement. The repeat measurements demonstrated high consistency, with ICC estimates ranging from 0.94 to 0.97. The individual ICC estimates and corresponding 95% confidence intervals are summarized in **Table 6**.

**Table 6:** Intraobserver reproducibility.

Parameter	ICC	95% CI	Interpretation
Tp-e	0.94	0.88–0.97	Excellent
QT	0.97	0.94–0.99	Excellent
JT	0.94	0.88–0.97	Excellent

## 4 Discussion

Assessment of ventricular repolarization provides valuable information on cardiac electrical changes accompanying thyroid hormone excess. In this study, repolarization was characterized using complementary ECG indices, including QT, QTc, JT, JTc, Tp-e, and Tp-e–derived measures, capturing different aspects of repolarization timing and heterogeneity. **Figure 4** schematically illustrates the ECG landmarks used to define the analyzed intervals and their relationship to ventricular depolarization and repolarization.

**Figure 4:**
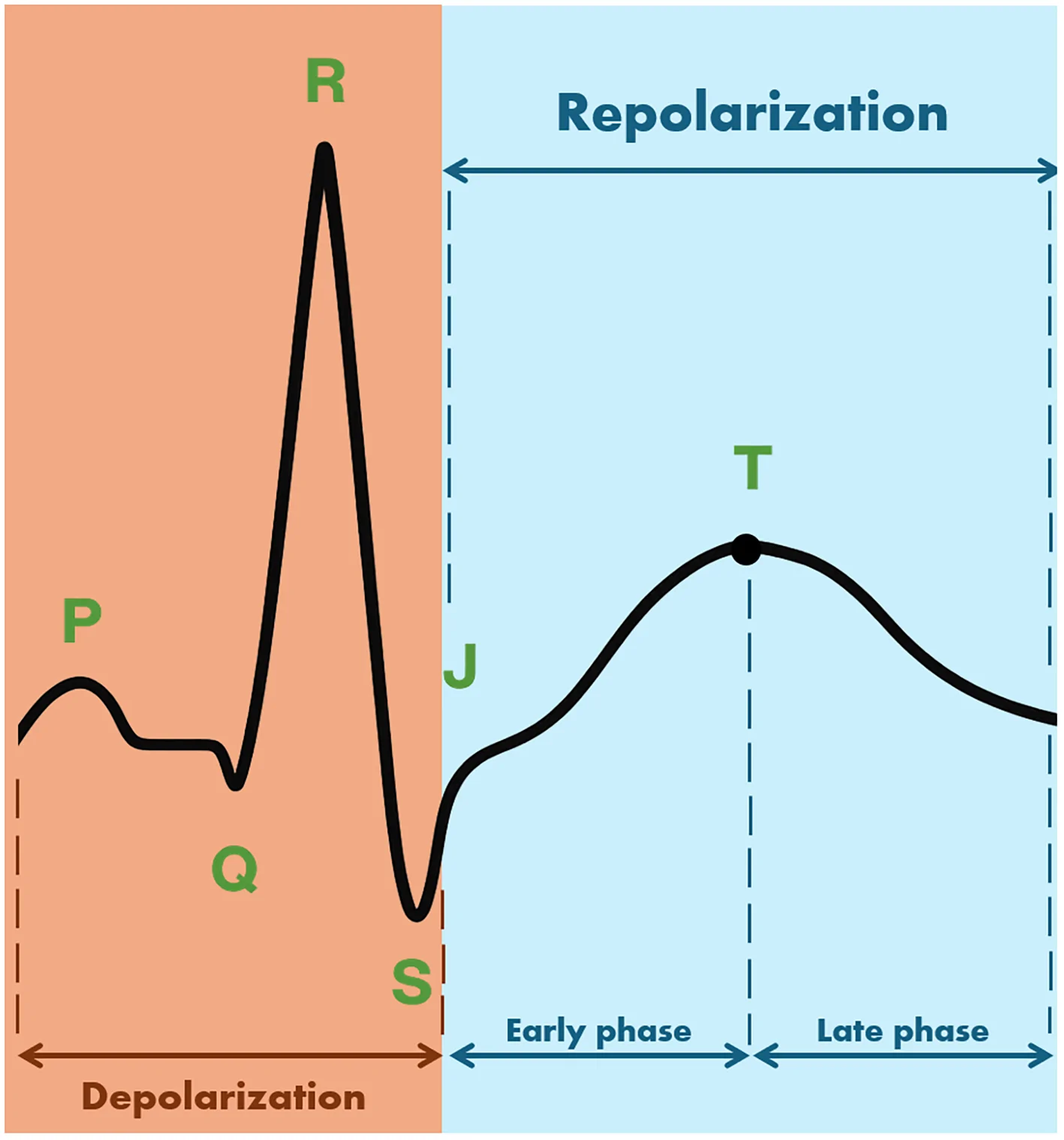
Schematic representation of ventricular depolarization and repolarization on a single-beat surface ECG. The P wave corresponds to atrial depolarization, while the QRS complex (Q, R, and S waves) represents ventricular depolarization. The end of the S wave marks the transition to the J point, which denotes the beginning of ventricular repolarization. The repolarization phase extends through the ST segment and culminates with the T wave. The colored areas illustrate the separation between depolarization (left panel) and repolarization (right panel), emphasizing the temporal boundary between these two electrophysiological processes.

The mechanisms underlying increased ventricular repolarization heterogeneity in Graves’ hyperthyroidism are likely multifactorial. Thyroid hormone excess enhances sympathetic activity and myocardial metabolism while affecting cardiac ion channel function and intracellular calcium handling, potentially producing regional differences in myocardial excitability and repolarization. These electrophysiological alterations may contribute to the increased repolarization dispersion observed in our study (6–8).

Among the investigated repolarization measures, Tp-e–based indices showed the most pronounced differences between patients with Graves’ hyperthyroidism and controls. Tp-e and Tp-e/QTc were significantly increased, while Tp-e/QT emerged as the strongest discriminator between the study groups and retained an independent association with Graves’ hyperthyroidism in the exploratory multivariable model. The performance of this ratio may be particularly relevant in the setting of increased heart rate, where interpretation of QTc can be substantially influenced by the applied correction formula. By relating repolarization dispersion to overall repolarization duration, Tp-e/QT may therefore provide information that is complementary to absolute interval measurements. These observations are supported by previous reports describing prolonged Tp-e and QTc intervals in Graves’ disease, as well as positive relationships between Tp-e and circulating FT3 and FT4 concentrations. Collectively, these findings further support the association between thyroid hormone excess and altered ventricular repolarization heterogeneity (9).

The clinical relevance of Tp-e–based measures has been investigated across a broad spectrum of cardiovascular disorders. Prolonged Tp-e and increased related indices have been associated with greater susceptibility to ventricular arrhythmias and sudden cardiac death. Such relationships have been reported in patients with premature ventricular contractions, structural cardiac diseases such as aortic stenosis, hypertrophic cardiomyopathy, and myocarditis, as well as in inherited arrhythmogenic conditions, including Brugada syndrome (10–20). Furthermore, Tp-e-based parameters have been associated with indices of cardiac remodeling, heart failure severity and coronary blood flow, supporting their role as non-invasive markers of ventricular repolarization abnormalities (14, 21, 22).

Importantly, alterations in ventricular repolarization are not limited to primary cardiovascular disorders. Abnormal repolarization indices have also been reported in systemic and metabolic conditions, including prediabetes, hypomagnesemia, and erectile dysfunction, suggesting that myocardial electrical properties may be influenced by extracardiac disturbances. This broader context is particularly relevant to endocrine disorders such as Graves’ disease, in which systemic thyroid hormone excess may similarly affect ventricular electrophysiology (23–25).

A modest increase in Bazett-corrected QT (QTcB) was observed in patients with Graves’ hyperthyroidism relative to controls. However, this difference needs to be considered in relation to the substantially higher heart rates in the Graves’ disease group, as Bazett’s formula tends to overcorrect the QT interval during tachycardia. Notably, no significant between-group difference was observed when QT was corrected using Fridericia’s formula. Taken together, these findings indicate that the observed elevation in QTcB may largely reflect the influence of heart rate and the correction method rather than a generalized increase in ventricular repolarization duration. The combination of shorter uncorrected QT intervals and increased dispersion-related indices suggests that Graves’ hyperthyroidism may alter the pattern of ventricular repolarization without uniformly extending its overall duration. This may reflect a preferential effect of thyroid hormone excess on repolarization heterogeneity across the ventricular myocardium. Such an interpretation is biologically plausible, as experimental and clinical studies indicate that thyroid hormones influence the expression and activity of cardiac ion channels, particularly potassium channels, potentially producing non-uniform changes in myocardial repolarization.

QTc prolongation may be either congenital or acquired. Congenital long QT syndrome is associated with an increased risk of malignant ventricular arrhythmias, syncope, cardiac arrest, and sudden cardiac death (26). Acquired QTc prolongation occurs in a wide range of clinical settings, including structural heart disease, electrolyte disturbances, systemic disorders, and drug therapy, particularly with antiarrhythmic agents and diuretics (27).

Even slight QTc prolongation may carry clinical significance, as it can provide a substrate for repolarization heterogeneity and an increased risk of ventricular arrhythmias, particularly in individuals with additional electrophysiological risk factors. In most studies, QTc prolongation has been associated with a higher risk of life-threatening arrhythmias or mortality. Alterations in ventricular repolarization increase its heterogeneity, facilitating the occurrence of torsade de pointes and other forms of ventricular tachyarrhythmias, which in some cases may lead to sudden cardiac death (28, 29). QTc prolongation observed in hyperthyroid patients is usually reversible and normalizes after achieving euthyroidism, as confirmed by previous clinical observations (30, 31).

In our cohort, the uncorrected JT interval was significantly shorter in patients with overt hyperthyroidism compared with controls, reflecting the overall acceleration of ventricular repolarization associated with sinus tachycardia. When corrected for heart rate using Bazett’s formula, the JTc interval was modestly prolonged in the hyperthyroid group, whereas no significant difference was observed for JTc calculated using Fridericia’s correction.

Given that Bazett-based correction is known to overestimate repolarization duration at higher heart rates, particularly in tachycardic states, the isolated prolongation of JTc Bazett should be interpreted with caution. The absence of corresponding prolongation in JTc Fridericia suggests that global ventricular repolarization duration is not uniformly prolonged in hyperthyroidism.

Currently, only a limited number of studies have examined the clinical significance of JT and JTc duration. Zulqarnain et al. demonstrated that both JT prolongation and shortening were associated with increased mortality, with JT showing greater prognostic value than QT among individuals with concomitantly prolonged JT and QT intervals (32, 33). As the JT interval excludes ventricular depolarization, it may more accurately reflect ventricular repolarization, particularly in patients with QRS prolongation. Experimental data further support the use of JT-based parameters as surrogate markers of proarrhythmic risk assessment (34).

The main finding of this study is that Graves’ hyperthyroidism is associated with altered ventricular repolarization, characterized primarily by increased heterogeneity rather than uniform prolongation of its overall duration. This pattern was reflected by consistent changes in Tp-e–based indices, whereas QTc and JTc showed less pronounced and correction-dependent differences. Among the evaluated parameters, Tp-e/QT demonstrated the strongest discriminatory performance, suggesting that relative measures of repolarization dispersion may provide additional information beyond global repolarization intervals. As Tp-e–based indices can be readily derived from a standard 12-lead ECG, their potential clinical relevance warrants further investigation. Prospective studies with longitudinal follow-up are needed to determine whether these measures are associated with ventricular arrhythmic outcomes and provide incremental prognostic information beyond established ECG parameters.

## 5 Limitations

This study has several limitations. First, arrhythmic outcomes were not assessed; therefore, Tp-e and related indices should be regarded as surrogate measures of ventricular repolarization heterogeneity rather than established predictors of arrhythmic risk. Second, the retrospective design prevented reliable determination of hyperthyroidism duration before ECG acquisition, precluding evaluation of potential time-dependent effects. Some variables included in model development were mathematically related, potentially introducing redundancy despite the applied variable-selection and multicollinearity procedures. Moreover, the relatively small sample size and lack of external validation limit model generalizability and do not exclude overfitting despite satisfactory internal bootstrap performance. The proposed model and nomogram should therefore be considered exploratory until validated in independent cohorts. Finally, the use of healthy controls may have enhanced the observed discriminatory performance, which could be lower in more heterogeneous clinical populations.

## 6 Conclusions and future directions

Graves’ hyperthyroidism is associated with measurable alterations in ventricular repolarization, characterized predominantly by increased repolarization heterogeneity rather than uniform prolongation of its overall duration. Tp-e and Tp-e–based indices showed the most consistent changes, with the Tp-e/QT ratio demonstrating the strongest discriminatory performance among the evaluated ECG parameters. In contrast, changes in QTc and JTc were less pronounced and varied according to the heart rate correction method, emphasizing the complexity of repolarization assessment in the setting of hyperthyroidism and increased heart rate. Tp-e–based measures may therefore provide complementary information on ventricular repolarization beyond conventional ECG intervals. However, their prognostic significance remains to be established. Future studies should include larger and more diverse patient cohorts and incorporate a broader range of ventricular repolarization indices. Such studies may help identify and validate reliable markers of electrical myocardial remodeling in Graves’ disease and determine whether these markers provide clinically relevant information for cardiovascular risk assessment and management. Prospective longitudinal studies comparing ECG parameters before treatment and after restoration of euthyroidism, and incorporating clinically relevant ventricular arrhythmic outcomes, are particularly warranted to establish the reversibility and prognostic significance of the observed electrical abnormalities.

## Data Availability

The raw data supporting the conclusions of this article will be made available by the authors, without undue reservation.

## Footnotes

1 This centering aligns coefficient estimates with the exploratory cut-off values identified in the preceding ROC analyses and facilitates interpretation of the model coefficients.
