# Supplementary material for: Cardiac electrical remodeling in Graves’ disease: insights from global ventricular repolarization indices
Source: Front Endocrinol (Lausanne). 2026 Sep 16;17:1979644. doi: 10.3389/fendo.2026.1979644 (PMC13623706; doi:10.3389/fendo.2026.1979644)
Supplement: Supplementary file 1 [file DataSheet1.pdf]

1. Statistical analysis

Statistical analyses were conducted with a predefined two-sided significance level (α) of 0.05. Normality was assessed via the Shapiro–Wilk test (Shapiro & Wilk, 1965) and Q–Q plots (Supplementary Figure QA-1); given anticipated non-Gaussian distributions, the Wilcoxon rank-sum test (Wilcoxon, 1945) served as the primary comparator for continuous variables between groups, with Pearson's chi-squared test applied to categorical variables (Pearson, 1900). Continuous variables were summarised as medians with interquartile ranges and 95% Wilcoxon-based confidence intervals; categorical variables as frequencies with Wilson score confidence intervals (Wilson, 1927). Permutation-based *p*-values (10,000 resamples; Hothorn et al., 2008) and Welch's *t*-tests were computed as sensitivity checks.

Within the OHT subgroup, associations between thyroid markers and the six dispersion parameters were quantified via Spearman's rank correlation (Spearman, 1904) with 95% percentile bootstrap confidence intervals (1,000 resamples; Efron & Tibshirani, 1993). No multiplicity adjustment was applied, following the Rothman (1990) rationale.

Diagnostic performance of each dispersion parameter was evaluated through receiver operating characteristic (ROC) analysis. The area under the curve (AUC) with 95% bootstrap confidence intervals (Hanley & McNeil, 1982) served as the primary discrimination metric. Optimal cutoffs were identified by maximising Youden's index (Youden, 1950), and performance was characterised by accuracy, sensitivity, specificity, and the full confusion matrix. Pairwise AUC comparisons employed DeLong's non-parametric test (DeLong et al., 1988).

To mitigate confounding, entropy balancing was implemented as a propensity score weighting procedure targeting the average treatment effect (ATE), equalising the first moments of sex, age, and BMI between groups without requiring a parametric propensity score model (Hainmueller, 2012). Balance was confirmed using standardised mean differences (SMD < 0.10; Austin, 2009), with weight stability evaluated through coefficients of variation, entropy values, and effective sample sizes (Zhao & Percival, 2017).

Multivariable logistic regression was employed to identify electrocardiographic variables independently associated with OHT. Variable selection was performed via LASSO penalised regression (α = 1, 10-fold cross-validation) incorporating entropy-balancing weights (Tibshirani, 1996; Friedman et al., 2010). Multicollinearity among selected predictors was resolved iteratively: at each step, the predictor with the highest variance inflation factor (VIF) exceeding 5 was removed until all VIF values fell below the threshold (Fox & Weisberg, 2019). Predictors were centred at their optimal ROC cutoffs or population medians. To address overfitting inherent to the

modest sample size, ridge penalisation was applied to the final model, with the optimal penalty algorithm maximising the corrected AIC (Harrell, 2015). The ridge-penalised model constituted the primary inferential framework; the unpenalised model was retained for reporting interpretable odds ratios with Wald-based inference. Internal validation (1,000 bootstrap resamples) yielded optimism-corrected discrimination (Somers' *D*xy, C-index), calibration (slope, *E*max), and overall performance metrics (Nagelkerke R^2, Brier score; Efron & Tibshirani, 1993; Harrell et al., 1996). A nomogram was constructed from the penalised model for clinical translation.

Pre-specified sensitivity analyses encompassed: alternative QTc correction formulae (Framingham, Hodges, Rautaharju) with AUC comparison via DeLong's test; Firth's bias-reduced logistic regression (Firth, 1993; Heinze & Schemper, 2002); permutation-based regression coefficients (5,000 resamples); leave-one-out influence diagnostics with Cook's distance (Cook, 1977); winsorised analysis (5th/95th percentiles); E-value computation for unmeasured confounding (VanderWeele & Ding, 2017); and comparison of weighted versus unweighted models.

1.1. Software and Reproducibility

Analyses were conducted using the R Statistical language (version 4.5.2; R Core Team, 2025) on Windows 10 Pro 64 bit (build 19045).

Data import and manipulation relied on *readxl* (version 1.4.5; Wickham & Bryan, 2025), dplyr (version 1.2.0; Wickham et al., 2026), *tidyr* (version 1.3.2; Wickham et al., 2025b), *stringr* (version 1.6.0; Wickham, 2025), *reshape2* (version 1.4.5; Wickham, 2007), and Matrix (version 1.7.4; Bates et al., 2025).

Descriptive statistics, confidence intervals, and summary tables were produced with *Hmisc* (version 5.2.5; Harrell, 2026) and *gtsummary* (version 2.5.0; Sjoberg et al., 2021). Permutation-based inference employed *coin* (version 1.4.3; Hothorn et al., 2006, 2008), while bootstrap resampling utilised *boot* (version 1.3.32; Canty & Ripley, 2025).

Receiver operating characteristic analysis and optimal cutpoint determination were performed with *pROC* (version 1.19.0.1; Robin et al., 2011) and *cutpointr* (version 1.2.1; Thiele & Hirschfeld, 2021). Propensity score weighting via entropy balancing and covariate balance assessment employed *WeightIt* (version 1.5.1; Greifer, 2025) and *cobalt* (version 4.6.2; Greifer, 2026).

Regression modelling encompassed LASSO penalised regression with *glmnet* (version 4.1.10; Friedman et al., 2010), variance inflation factor diagnostics with *car* (version 3.1.5; Fox & Weisberg, 2019), and ridge-penalised logistic regression with bootstrap validation, calibration,

and nomogram construction through *rms* (version 8.1.1; Harrell, 2026) and *survival* (version 3.8.3; Therneau, 2024). Sensitivity analyses were supported by *logistf* (version 1.26.1; Heinze et al., 2025) for Firth's bias-reduced logistic regression and *EValue* (version 4.1.4; VanderWeele & Ding, 2017) for unmeasured confounding assessment.

Visualisation utilised *ggplot2* (version 4.0.2; Wickham, 2016), *cowplot* (version 1.2.0; Wilke, 2025), *ggrepel* (version 0.9.7; Slowikowski, 2026), *corrplot* (version 0.95; Wei & Simko, 2024), and *scales* (version 1.4.0; Wickham et al., 2025a). Tabular and document export was facilitated by *flextable* (version 0.9.11; Gohel & Skintzos, 2026) and *officer* (version 0.7.3; Gohel et al., 2026).

2. **ECG measurement methodology**

The Tp-e interval was measured from the peak to the end of the T-wave, primarily in precordial lead V5. The peak of the T wave was identified as the point of maximum amplitude of the T wave. The end of the T wave was determined using the tangent method - intersection of the tangent to the steepest slope of the terminal limb of the T wave with the isoelectric baseline (Fig.1). Leads V4 or V6 were used when the T-wave amplitude in V5 was insufficient (< 1.5 mm) or morphology was unclear(5). The mean of three consecutive beats was calculated for each patient. Values were rounded to the nearest whole number.

The RR interval was measured as the interval between the peaks of two consecutive R waves on the ECG trace, representing one complete cardiac cycle. Measurements were performed in sinus rhythm and expressed in milliseconds (Fig.1). Heart rate (HR) was calculated from the RR interval measured using the formula HR = 60,000 / RR. Values were rounded to the nearest whole number.

The QT interval was measured from the onset of the QRS complex (defined as the first deflection from the isoelectric line) to the end of the T wave (defined as above), primarily in the same lead in which the Tp–e interval was assessed (Fig.1). The mean value from three consecutive beats was calculated for each patient. Values were rounded to the nearest whole number. Corrected QT interval (QTc) was determined using Bazett's (QTcB) and Fridericia's formulas (QTcF) to account for heart rate variability, as sinus tachycardia is common in hyperthyroidism(6). In the main analysis, QTcF was used as it provides a more accurate correction at higher heart rates, which are characteristic of the studied population.

The Tp–e/QT and Tp–e/QTc ratios were calculated as the quotient of the Tp–e interval and the QT or QTcF, using the previously described methodology.

The JT interval was measured as the time from the J point (defined as the junction between the end of the QRS complex and the beginning of the ST segment) to the end of the T wave (defined as above), primarily in the same lead in which the Tp–e interval was assessed (Fig.1). The mean of three consecutive beats was calculated for each patient. Values were rounded to the nearest whole number.

JT was then corrected for heart rate using Bazett's ($JTcB = JT/\sqrt{RR}$) and Fridericia's ($JTcF = JT/\sqrt[3]{RR}$) formulas. RR intervals were recorded in milliseconds and were converted to seconds before applying the correction formulas to ensure mathematical consistency.

3. Multivariable Predictive Analysis

3.1. Propensity Score Weighting and Covariate Balance

Entropy balancing was applied to estimate weights targeting the average treatment effect in the population, equalising the first moments of sex, age, and body mass index between the OHT and Control groups. Weight diagnostics (Supplementary Table S.2) confirmed a narrow distribution around unity in both groups (coefficient of variation ≤ 0.14; range: 0.781–1.312), with no zero weights and negligible effective sample size reduction (from 55 to 54.1 in OHT; from 42 to 41.2 in controls), indicating that the balancing procedure preserved virtually all statistical information while achieving the desired covariate adjustment.

Prior to weighting, a mild imbalance was present for sex (SMD = 0.115, marginally exceeding the 0.10 threshold), reflecting the higher proportion of females in the OHT group – a pattern consistent with the epidemiological sex distribution of hyperthyroidism. Age and BMI exhibited smaller pre-weighting differences (SMD = −0.084 and 0.046, respectively), both within acceptable limits. Following entropy balancing, exact equilibrium was attained across all three covariates, with adjusted standardised mean differences of precisely zero (Supplementary Table S.3). The trajectory from pre-weighting imbalance to perfect post-weighting equilibrium is visualised in Supplementary Figure S.1, where directional arrows trace each covariate's convergence toward the balance threshold.

Positivity – a prerequisite for valid causal inference under the entropy balancing framework – was confirmed graphically (Supplementary Figure QA-2). The propensity score distribution exhibited complete overlap between groups, with the full range confined to 0.43–0.63, well within the 0.05–0.95 boundaries beyond which positivity violations would be indicated. Group medians were

virtually identical (0.60 in both), reflecting the demographic comparability established in the first results section and confirming that every individual in the analytic cohort occupies a region of common support where both treatment states are empirically represented.

These results establish that the entropy-balanced weights are stable, comprehensive, and methodologically sound, thereby providing a credible foundation for the weighted logistic regression analyses reported in the following section.

3.2. Multivariable Logistic Regression

Predictor selection commenced with seven candidate variables – the six dispersion parameters plus heart rate – subjected to LASSO penalised logistic regression incorporating entropy-balancing weights. The shrinkage pathway (Supplementary Figure S.2) demonstrates the progressive attenuation of coefficients toward zero as regularisation intensifies, with five predictors (QTd, QTcBd, JTcFd, Tp-ed, HR) retained at λ_{min} and four (QTcBd, JTcFd, Tp-ed, HR) at the more parsimonious λ_{1SE}. The cross-validation deviance profile (Supplementary Figure S.3) confirms the stability of these selection points. Iterative variance inflation factor screening subsequently identified substantial multicollinearity between QTd and QTcBd (VIF > 27), prompting removal of QTd. Following this resolution, all retained predictors exhibited VIF values below 1.42, confirming the absence of collinearity in the final predictor set.

The entropy-weighted logistic regression model incorporating QTcBd, JTcFd, Tp-ed, and heart rate is presented in Table A.1.

Predictor	β	SE	OR [95% CI]	Wald Z	p
**Intercept**	0.2140	0.2835	1.24 [0.71–2.16]	0.75	.450
**QTc Dispersion – Bazett (QTcBd), centred at 37.9 ms**	0.0454	0.0244	1.05 [1.00–1.10]	1.86	.063
**JTc Dispersion – Fridericia (JTcFd), centred at 32.7 ms**	0.0318	0.0218	1.03 [0.99–1.08]	1.46	.145
**Tp–e Dispersion (Tp-ed), centred at 29.0 ms**	0.0609	0.0289	1.06 [1.00–1.12]	2.11	.035
**Heart Rate (HR), centred at 90.4 bpm**	0.0152	0.0120	1.02 [0.99–1.04]	1.27	.205

Table A.1. *Multivariable Logistic Regression Coefficients for the Entropy-Weighted Model Assessing Associations with Overt Hyperthyroidism: Tp-e Dispersion Emerges as the Only Individually Significant Variable Associated with Overt Hyperthyroidism, While Overall Model Performance Is Sustained by Ridge Penalisation (N = 97)*

Note. Primary framework: ridge penalisation (penalty = 3.0, pentrace/AICc). LR χ^2 = 38.34 (effective df = 3, p < .001; nominal df = 4, reduced by ridge penalisation). Apparent C-index = 0.874; Nagelkerke R^2 = 0.417. Optimism-corrected calibration slope = 1.04 (B = 1,000). All VIF < 5 (max = 1.41); EPV = 13. Coefficients from the unpenalised model for interpretability; penalised coefficients are shrunken toward zero. β = log-odds coefficient; OR = odds ratio; CI = confidence interval.

Among the four predictors, Tp-e dispersion was the sole variable attaining individual statistical significance (OR = 1.06 per ms increase above 29.0 ms, 95% CI [1.00–1.12], *p* = .035), corresponding to a 6% increase in the odds of belonging to the OHT group. QTcBd approached significance (OR = 1.05 per ms above 37.9 ms, 95% CI [1.00–1.10], *p* = .063), while JTcFd (OR = 1.03, *p* = .145) and heart rate (OR = 1.02 per bpm above 90.4, *p* = .205) contributed to joint model performance without reaching individual thresholds. Crucially, the overall model was highly significant (LR χ^2 = 38.34, effective df = 3, *p* < .001), with an apparent C-index of 0.874 and Nagelkerke R^2 of 0.417, confirming that the four predictors collectively capture substantial information about OHT status despite the modest individual effect sizes.

To address the overfitting inherent to the modest sample size (EPV = 13), ridge penalisation was applied with an optimal penalty of 3.0 identified via pentrace/AICc maximisation. The penalised model achieved an optimism-corrected calibration slope of 1.04 – virtually indistinguishable from the ideal value of 1.0 – compared with 0.64 for the unpenalised counterpart, representing a decisive remediation of overfitting without meaningful loss in discriminatory capacity.

A nomogram derived from the penalised model (Supplementary Figure S.5) is presented to facilitate visualisation of the relative contribution of model variables and the estimated probabilities generated within the study cohort. The concordance of odds ratios across four analytical approaches – primary entropy-weighted GLM, Firth's bias-reduced regression, winsorised analysis, and unweighted GLM – is visualised in the forest plot (Supplementary Figure S.7), demonstrating consistent directionality and comparable magnitudes for all four predictors across all models. Firth's regression (Supplementary Table S.5) confirmed that Tp-ed retained its significance (OR = 1.06, *p* = .036), while all remaining predictors exhibited near-identical point estimates, ruling out quasi-complete separation or small-sample artefacts as drivers of the primary findings. The weighted versus unweighted comparison (Supplementary Table S.6) yielded closely aligned odds ratios (C-index: 0.870 vs. 0.888), confirming that the entropy-balancing adjustment did not distort the inferential conclusions. The E-value for the significant Tp-ed association (Supplementary Table S.7) was 1.21, indicating that an unmeasured confounder associated with both hyperthyroidism and Tp-e dispersion at a risk ratio of at least 1.21 – beyond the adjustment for sex, age, and BMI – would be required to explain away the observed effect entirely. Leave-one-out influence diagnostics (Supplementary Figure S.9) confirmed that no single observation

exceeded the conventional Cook's distance threshold of 4/N, indicating that the model estimates are not disproportionately driven by any individual case.

3.3. Internal Validation and Calibration

Internal validation of the ridge-penalised logistic regression model was conducted through 1,000 bootstrap resamples, with the complete set of optimism-corrected performance indices reported in Table A.2.

Index	Original	Training	Test	Optimism	Corrected
**Somers' Dxy**	0.747	0.814	0.770	0.045	0.703
**Nagelkerke R^2**	0.417	0.479	0.418	0.060	0.357
**Calibration Intercept**	0.000	0.000	−0.021	0.021	−0.021
**Calibration Slope**	1.000	1.000	1.039	−0.039	1.039
**Max. Calibration Error (Emax)**	0.000	0.000	0.073	−0.073	0.073
**Discrimination Index (D)**	0.385	0.437	0.364	0.073	0.312
**Unreliability Index (U)**	−0.021	−0.021	−0.002	−0.018	−0.002
**Quality Index (Q = D – U)**	0.406	0.457	0.366	0.091	0.314
**Brier Score**	0.151	0.124	0.138	−0.014	0.165
**Gini Concentration (g)**	1.760	1.952	2.057	−0.104	1.864
**Explained Variation (gp)**	0.308	0.319	0.328	−0.009	0.316

Table A.2. *Bootstrap Validation of the Ridge-Penalised Logistic Regression Model.*

Note. Ridge penalty = 3.0 (pentrace/AICc). Optimism = average training–test difference; corrected = adjusted for overfitting. Slope = 1.04 (≈ ideal). Corrected C-index = 0.5 × (1 + Dxy) = 0.851.

The model demonstrated good discrimination within the study cohort. The optimism-corrected Somers' D_{xy} was 0.703, corresponding to a corrected C-index of 0.851 – indicating that in 85% of randomly selected OHT–Control pairs, the model correctly assigns a higher predicted probability to the hyperthyroid individual. The optimism in D_{xy} was modest (0.045), confirming that the ridge penalty effectively constrained the degree to which the model capitalised on sample-specific patterns. Nagelkerke R^2 declined from 0.417 (apparent) to 0.357 (corrected), representing a 14%

reduction attributable to optimism – a moderate and expected degree of shrinkage given the EPV of 13.

The calibration metrics constitute the most consequential validation finding. The optimism-corrected calibration slope was 1.039 – virtually identical to the ideal value of 1.0 – signifying that predicted probabilities generated by the penalised model correspond faithfully to observed event rates across the full spectrum of risk. The maximum absolute calibration error (E_{max}) was 0.073, indicating that the largest discrepancy between predicted and observed probabilities at any point along the calibration curve did not exceed 7.3 percentage points. The calibration intercept remained near zero (−0.021), confirming the absence of systematic over- or under-prediction. Graphical calibration assessment (Supplementary Figure S.4) corroborates these metrics: the bias-corrected curve closely tracks the ideal diagonal, with no appreciable deviation in either the low- or high-probability regions. The Brier score of 0.165 further attests to the overall prediction accuracy, combining both discrimination and calibration into a single measure of overall model performance.

The three-model comparison (Supplementary Table S.9) contextualises these results against the unpenalised and parsimonious alternatives. The unpenalised model, despite marginally higher apparent discrimination (C-index 0.849), exhibited a calibration slope of only 0.641 – a value indicating that its predicted probabilities are substantially miscalibrated and excessively extreme. The parsimonious two-predictor model (Tp-ed + QTcBd) achieved reasonable calibration (slope = 0.936) but at the cost of reduced discrimination (C-index 0.823). The ridge-penalised model thus occupies the optimal position in the bias–variance trade-off: it preserves the full discriminatory information contributed by four predictors while delivering calibration accuracy that matches, and in practical terms exceeds, the parsimonious alternative.

3.4. Supplementary tables

Parameter	n	Median (Q1–Q3), [Min, Max]
**Free triiodothyronine (FT3, pg/mL)**	55	8.52 (5.30–17.28), [2.21, 27.59]
**Free thyroxine (FT4, ng/dL)**	55	3.28 (2.43–4.75), [1.21, 17.00]
**Creatinine (mg/dL)**	53	0.65 (0.47–0.80), [0.32, 2.30]
**eGFR (mL/min/1.73 m^2)**	52	110.50 (96.00–119.50), [21.00, 124.00]
**Haemoglobin (g/dL)**	53	12.90 (12.10–14.10), [10.30, 16.60]

**Haematocrit (%)**	53	38.00 (35.10–40.80), [30.60, 49.70]
**Sodium (mmol/L)**	48	139.50 (138.00–141.00), [135.00, 148.00]
**Potassium (mmol/L)**	48	4.20 (4.00–4.40), [3.50, 4.90]
**Chloride (mmol/L)**	48	106.00 (103.50–107.50), [99.00, 113.00]
**Calcium (mg/dL)**	22	9.40 (9.21–9.76), [8.84, 10.50]

Table S.1. *Biochemical and Haematological Characterisation of the OHT Subgroup: Profoundly Deranged Thyroid Function with Preserved Renal, Haematological, and Electrolyte Homeostasis (N = 55)*

Note. n = available observations. Censored values (e.g., anti-TPO > 1,300) replaced by boundary. eGFR = estimated glomerular filtration rate.

Group	N	W min	W max	W mean	CV	MAD	Zero wt.	ESS
**Healthy Controls**	42	0.781	1.131	1.000	0.141	0.131	0	41.2
**Overt Hyperthyroidism**	55	0.873	1.312	1.000	0.128	0.101	0	54.1

Table S.2. *Entropy Balancing Weight Diagnostics: Narrow Distribution Around Unity, Zero Excluded Observations, and Minimal Effective Sample Size Reduction Confirm Stable Weighting (N = 97)*

Note. Entropy balancing targeting ATE, equalising first moments of sex, age, and BMI. CV = coefficient of variation; MAD = mean absolute deviation; ESS = effective sample size; Zero wt. = number of zero weights. All weights non-negative; ESS reduction < 2%.

Table S.3. *Standardised Mean Differences Before and After Entropy Balancing: Perfect Post-Weighting Equilibrium Achieved on All Three Covariates (|SMD| = 0.00)*

Covariate	Type	SMD (unadjusted)	SMD (adjusted)	Balance status
**Sex (proportion male)**	Binary	0.115	0.000	Balanced
**Age (years)**	Continuous	−0.084	0.000	Balanced
**Body Mass Index (kg/m^2)**	Continuous	0.046	0.000	Balanced

Table S.3. *Standardised Mean Differences Before and After Entropy Balancing: Perfect Post-Weighting Equilibrium Achieved on All Three Covariates (|SMD| = 0.00)*

Note. SMD = standardised mean difference (pooled SD for continuous; appropriate standardisation for binary). Threshold: |SMD| < 0.10. Sex exhibited mild pre-weighting imbalance (0.12 > 0.10); all covariates attained exact balance (0.00) post-weighting.

Correction Formula	AUC [95% CI]
**Bazett**	0.510 [0.425–0.595]
**Fridericia**	0.509 [0.424–0.594]
**Framingham**	0.732 [0.654–0.810]
**Hodges**	0.732 [0.654–0.810]
**Rautaharju**	0.732 [0.654–0.810]

Table S.4. *Sensitivity Analysis: AUC of QTc Dispersion Across Five Rate-Correction Formulae – Framingham, Hodges, and Rautaharju Outperform Bazett and Fridericia When Derived from the Detailed QT Analysis Sheet*

Note. AUC with 95% bootstrap CI. Bazett/Fridericia values differ from Table 2 because they derive from the detailed QT sheet (recomputed max/min per formula independently). Linear correction methods (Framingham, Hodges, Rautaharju) produce identical dispersion when applied to the same max/min QT values.

Predictor	β	SE	OR [95% CI]	p
**Intercept**	0.210	0.270	1.23 [0.72–2.17]	.445
**QTc Dispersion – Bazett (QTcBd)**	0.035	0.023	1.04 [0.99–1.09]	.139
**JTc Dispersion – Fridericia (JTcFd)**	0.030	0.020	1.03 [0.99–1.07]	.136
**Tp–e Dispersion (Tp-ed)**	0.055	0.027	1.06 [1.00–1.12]	.036
**Heart Rate (HR)**	0.020	0.012	1.02 [1.00–1.05]	.073

Table S.5. *Firth's Bias-Reduced Logistic Regression: Coefficient Estimates and Odds Ratios Concordant with the Primary Model, Confirming Robustness to Potential Separation and Small-Sample Bias*

Note. Profile penalised-likelihood CIs replace Wald-based CIs. Tp-ed retains significance ($p = .036$), consistent with Table 3. Firth's method addresses bias from quasi-complete separation (Firth, 1993; Heinze & Schemper, 2002).

Model	Predictor	OR [95% CI]	p
**Weighted (entropy-balanced)**	QTcBd	1.05 [1.00–1.10]	.063
	JTcFd	1.03 [0.99–1.08]	.145
	Tp-ed	1.06 [1.00–1.12]	.035
	HR	1.02 [0.99–1.04]	.205
**Unweighted (no balancing)**	QTcBd	1.04 [0.99–1.09]	.131
	JTcFd	1.03 [0.99–1.08]	.119
	Tp-ed	1.06 [1.00–1.13]	.035
	HR	1.02 [1.00–1.05]	.078

Table S.6. *Weighted Versus Unweighted Model Comparison: Consistent Direction and Magnitude of Odds Ratios with Comparable Discriminatory Performance (C-Index: 0.870 vs. 0.888)*

Note. C-index: Weighted = 0.870; Unweighted = 0.888. Tp-e dispersion remained the sole individually significant predictor in both models ($p = .035$). The unweighted model's marginally higher C-index reflects absence of balancing adjustment rather than superior capacity.

Predictor	OR	E-value (point)	E-value (CI bound)
**Tp–e Dispersion (Tp-ed)**	1.06	1.21	1.05

Table S.7. *E-Value Assessment of Vulnerability to Unmeasured Confounding: The Significant Association of Tp-e Dispersion Would Require an Unmeasured Confounder of Modest Strength (E-Value = 1.21) to Be Fully Explained Away*

Note. The E-value quantifies the minimum strength of association (risk-ratio scale) an unmeasured confounder would need with both exposure and outcome, beyond measured covariates, to reduce the observed estimate to the null (VanderWeele & Ding, 2017). The CI-bound E-value of 1.05 represents the threshold to shift the confidence interval to include 1.0.

Parameter 1	Parameter 2	AUC 1	AUC 2	Z	p	Sig.
QTd	QTcBd	0.727	0.804	−4.277	< .001	Yes
QTd	QTcFd	0.727	0.781	−4.339	< .001	Yes
QTd	JTd	0.727	0.765	−0.731	.465	–
QTd	JTcFd	0.727	0.813	−1.761	.078	–
QTd	Tp-ed	0.727	0.725	0.027	.979	–
QTcBd	QTcFd	0.804	0.781	3.167	.002	Yes
QTcBd	JTd	0.804	0.765	0.819	.413	–
QTcBd	JTcFd	0.804	0.813	−0.193	.847	–
QTcBd	Tp-ed	0.804	0.725	1.266	.205	–
QTcFd	JTd	0.781	0.765	0.312	.755	–
QTcFd	JTcFd	0.781	0.813	−0.713	.476	–
QTcFd	Tp-ed	0.781	0.725	0.873	.383	–
JTd	JTcFd	0.765	0.813	−4.072	< .001	Yes
JTd	Tp-ed	0.765	0.725	0.570	.568	–
JTcFd	Tp-ed	0.813	0.725	1.283	.200	–

Table S.8. *DeLong Pairwise AUC Comparisons: QTcBd and QTcFd Demonstrate Significantly Higher Discriminatory Capacity Than QTd, While JTcFd Significantly Outperforms JTd (N = 97)*

Note. DeLong's non-parametric test for correlated ROC curves. Z = DeLong Z-statistic. Significant pairs ($p < .05$) marked "Yes." All AUC values from the same cohort; correlations between paired curves accounted for in the test statistic.

Model	k	EPV	Dxy (orig.)	Dxy (corr.)	C (corr.)	R^2 (corr.)	Slope	Emax	Brier
**Unpenalised (full)**	4	13	0.751	0.699	0.849	0.253	0.641	0.163	0.162
**Ridge-penalised (penalty = 3.0)**	4	13	0.747	0.703	0.851	0.357	1.039	0.073	0.165
**Parsimonious (Tp-ed + QTcBd)**	2	27	0.667	0.647	0.823	0.373	0.936	0.081	0.184

Table S.9. *Three-Model Comparison: Ridge Penalisation (Penalty = 3.0) Achieves Near-Ideal Calibration (Slope = 1.04) While Preserving the Highest Optimism-Corrected C-Index (0.851) Among Candidate Models*

Note. All indices optimism-corrected (B = 1,000; N = 97). k = predictors; EPV = events per variable. Unpenalised: slope = 0.64 (substantial overfitting). Ridge: slope = 1.04 (ideal). Parsimonious: slope = 0.94 with reduced C-index. Ridge selected as the primary framework.

Thyroid Marker	ECG Parameter	ρ [95% CI]	p
FT3 (pg/mL)	QTd (ms)	0.153 [−0.123, 0.444]	.266
	QTcBd (ms)	0.163 [−0.129, 0.430]	.233
	QTcFd (ms)	0.152 [−0.112, 0.424]	.269
	JTd (ms)	0.238 [−0.015, 0.450]	.080
	JTcFd (ms)	0.258 [−0.004, 0.480]	.057
	Tp-ed (ms)	0.021 [−0.237, 0.288]	.880
FT4 (ng/dL)	QTd (ms)	−0.072 [−0.343, 0.192]	.603
	QTcBd (ms)	0.063 [−0.203, 0.312]	.650
	QTcFd (ms)	0.010 [−0.256, 0.276]	.944
	JTd (ms)	0.109 [−0.137, 0.357]	.428
	JTcFd (ms)	0.197 [−0.034, 0.433]	.150
	Tp-ed (ms)	−0.124 [−0.378, 0.133]	.366
TSH (μIU/mL)	QTd (ms)	−0.049 [−0.300, 0.214]	.722
	QTcBd (ms)	−0.145 [−0.415, 0.098]	.289
	QTcFd (ms)	−0.117 [−0.375, 0.130]	.395
	JTd (ms)	−0.112 [−0.363, 0.159]	.416
	JTcFd (ms)	−0.161 [−0.413, 0.095]	.240
	Tp-ed (ms)	0.071 [−0.197, 0.325]	.606

Table S.10. *Spearman Rank Correlations Between Thyroid Function Markers and Ventricular Repolarization Dispersion Parameters: Limited Monotonic Associations Within the Hyperthyroidism Subgroup (N = 55)*

Note. ρ = Spearman rank correlation coefficient; 95% CI = percentile bootstrap CI (1,000 resamples). No multiplicity adjustment applied (Rothman, 1990). All correlations were weak and non-significant ($p > .05$), indicating limited monotonic dependence between thyroid hormones and dispersion indices within this subgroup.

2. Supplementary Figures

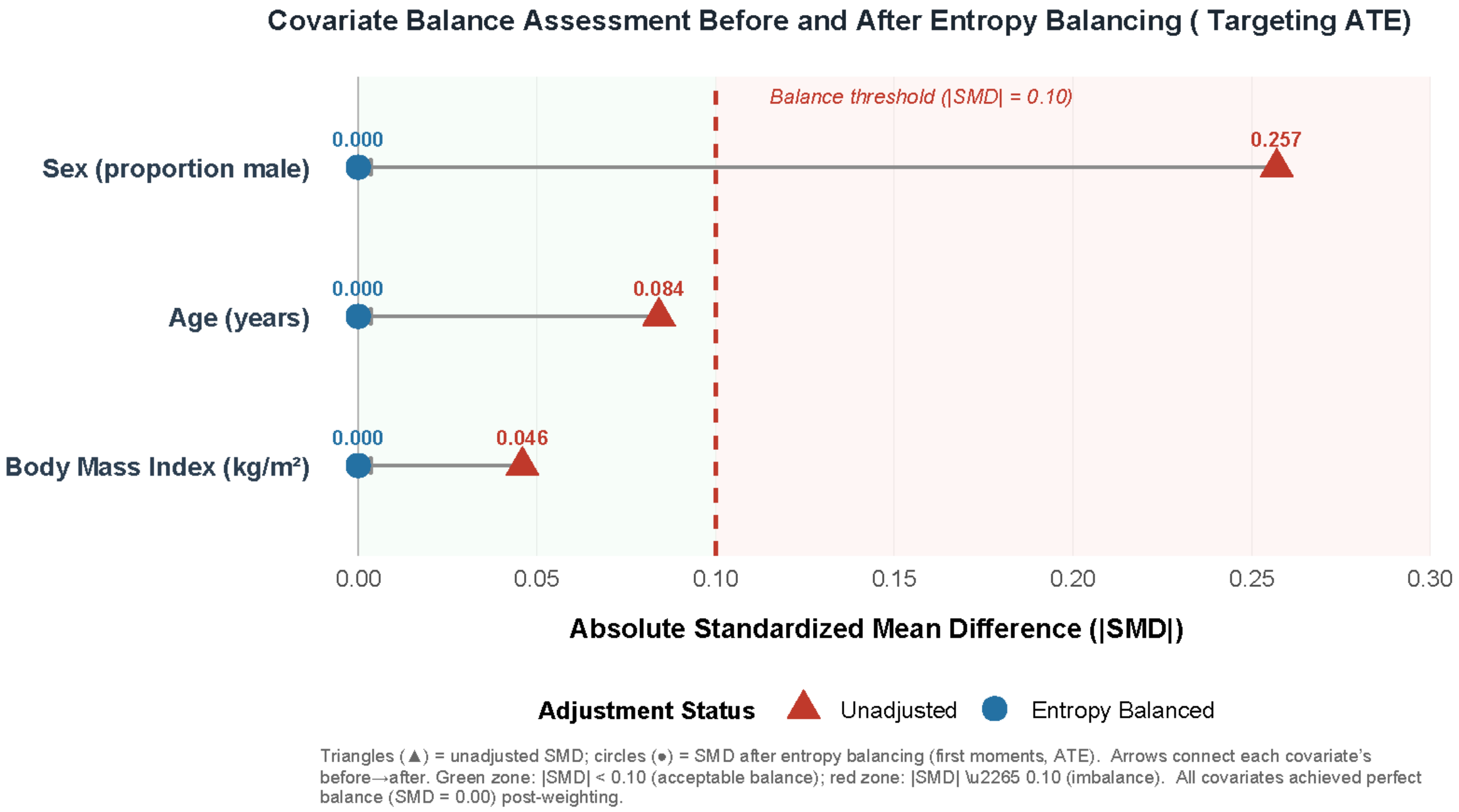

Figure S.1. *Covariate Balance Assessment Before and After Entropy Balancing: Arrows Depict the Trajectory from Pre-Weighting Imbalance to Perfect Post-Weighting Equilibrium on Sex, Age, and BMI (|SMD| = 0.00)*

Note. Triangles (▲) = unadjusted SMD; circles (●) = SMD after entropy balancing (first moments, ATE). Green zone: |SMD| < 0.10 (acceptable balance); red zone: |SMD| ≥ 0.10 (imbalance). Numeric values annotated beside each marker. SMD = standardised mean difference.

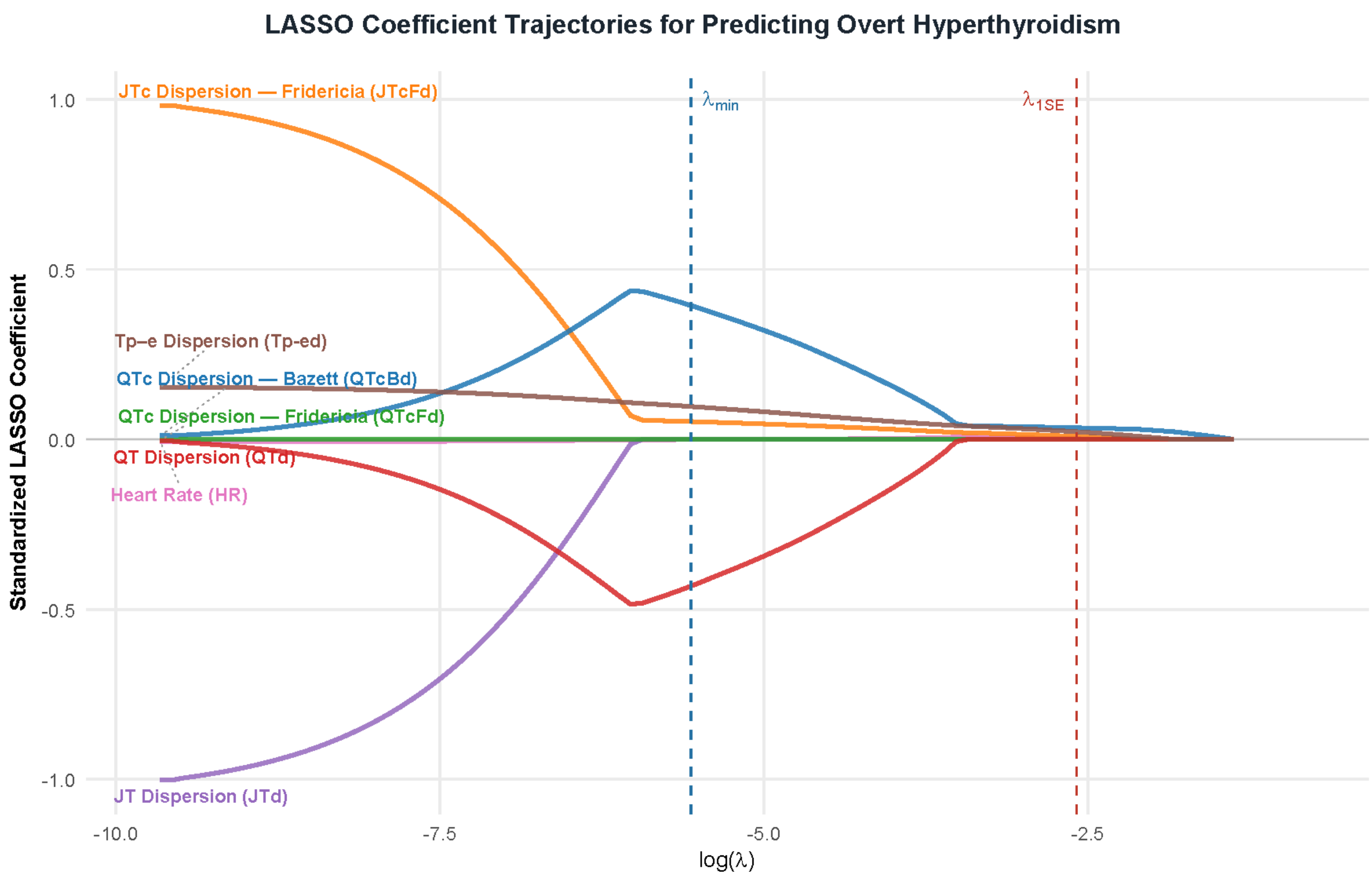

Figure S.2. *LASSO Coefficient Trajectories for Seven Candidate Predictors of Overt Hyperthyroidism: Five Predictors (QTd, QTcBd, JTcFd, Tp-ed, HR) Retained at λ_min, with QTcFd and JTd Shrunk to Zero; QTd Subsequently Removed via Iterative VIF Resolution*

Note. LASSO (L1) penalised logistic regression with entropy-balancing weights (binomial family, 10-fold CV, standardised coefficients). Dashed lines: λ at minimum CV deviance (blue) and one-standard-error rule (red). Labels identify each predictor at the least-regularised endpoint.

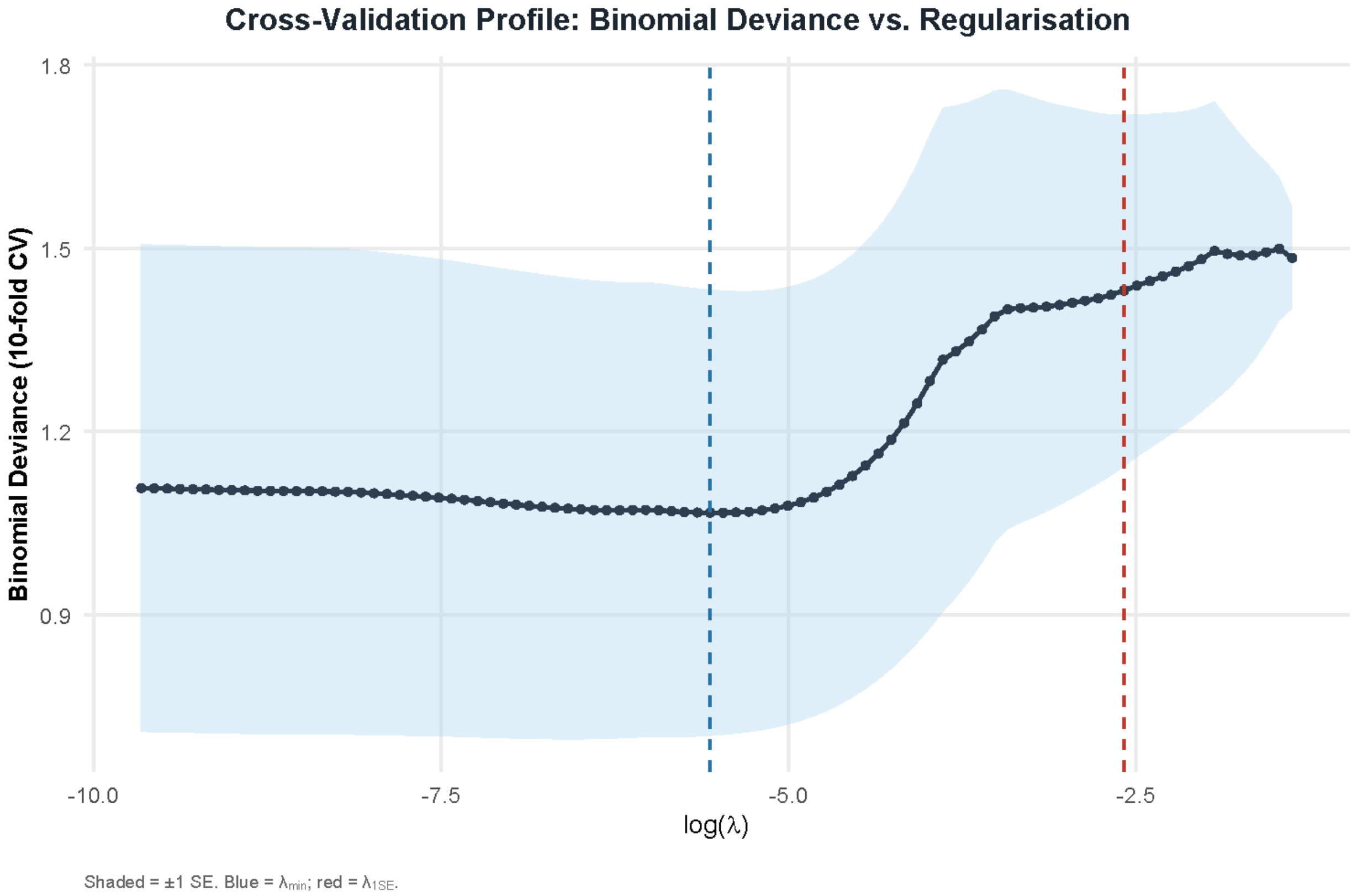

Figure S.3. *Cross-Validation Profile of Binomial Deviance Versus log(λ) Identifies Optimal Regularisation at λ_{min} (5 Predictors) and the Parsimonious λ_{1SE} (4 Predictors)*

Note. Shaded band = ±1 SE of the 10-fold cross-validated deviance. Dashed vertical lines mark λ_min (blue) and λ_1SE (red) with the corresponding number of active predictors annotated.

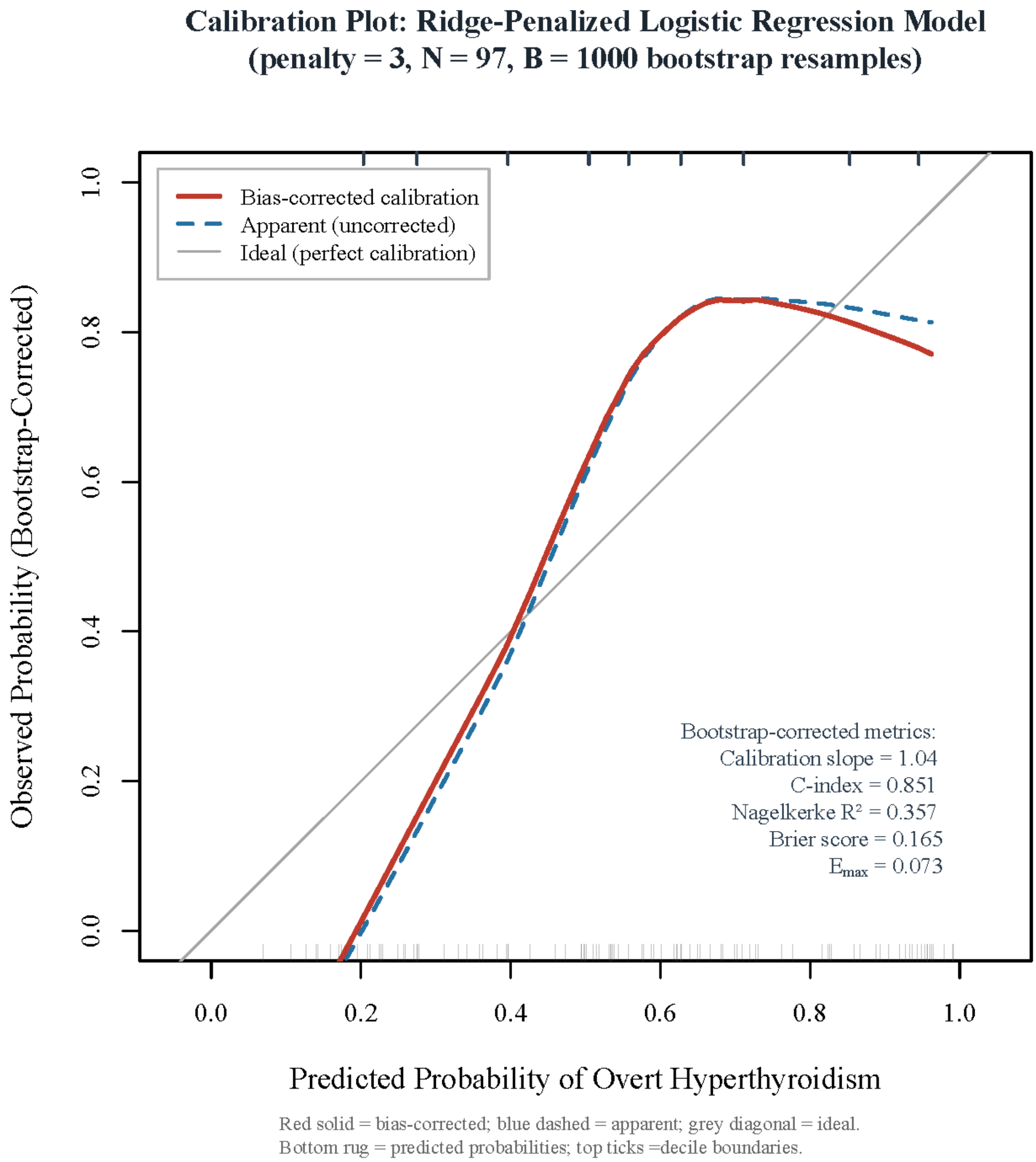

Figure S.4. *Calibration Plot of the Ridge-Penalised Logistic Regression Model: Bias-Corrected Curve Closely Tracks the Ideal Diagonal, with Optimism-Corrected Slope = 1.04 and C-Index = 0.851 (B = 1,000; N = 97)*

Note. Red solid = bias-corrected calibration (1,000 bootstrap resamples); blue dashed = apparent (uncorrected); grey diagonal = ideal (perfect calibration). Bottom rug marks = distribution of individual predicted probabilities; top ticks = decile boundaries. Bootstrap-corrected metrics annotated within the panel: calibration slope, C-index, Nagelkerke R^2, Brier score, and E_max.

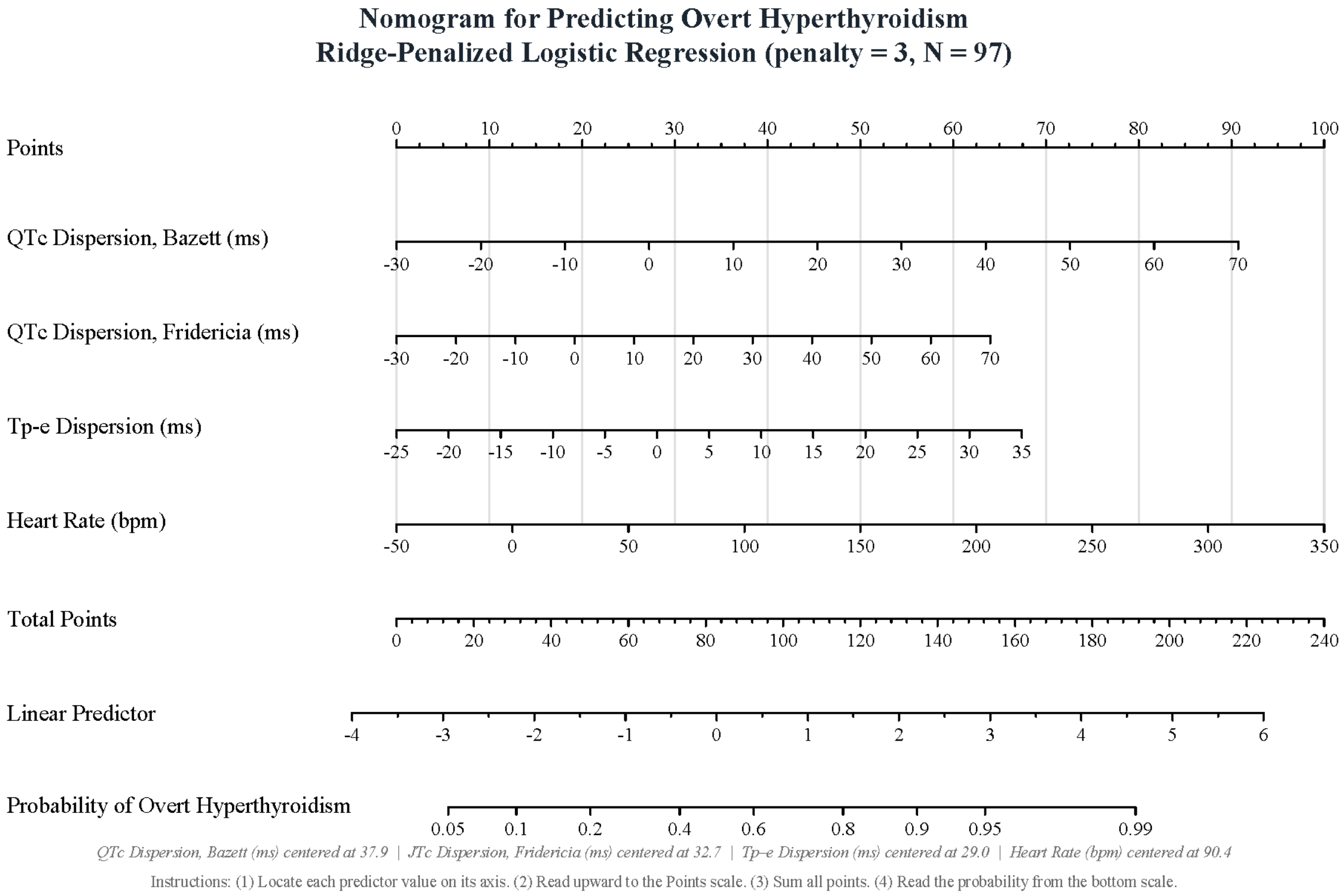

Figure S.5. *Nomogram Derived from the Ridge-Penalised Model illustrates the contribution of model variables to estimated probabilities within the study cohort Using QTcBd, JTcFd, Tp-ed, and Heart Rate (Penalty = 3.0; N = 97)*

Note. Instructions: (1) Locate each predictor value on its respective axis; (2) read upward to the Points scale; (3) sum all points to obtain Total Points; (4) read the corresponding probability from the bottom scale. Predictors are centred at their Youden-optimised cutoffs or population medians (values annotated below the panel). Ridge penalty = 3.0 (selected via pentrace/AICc).

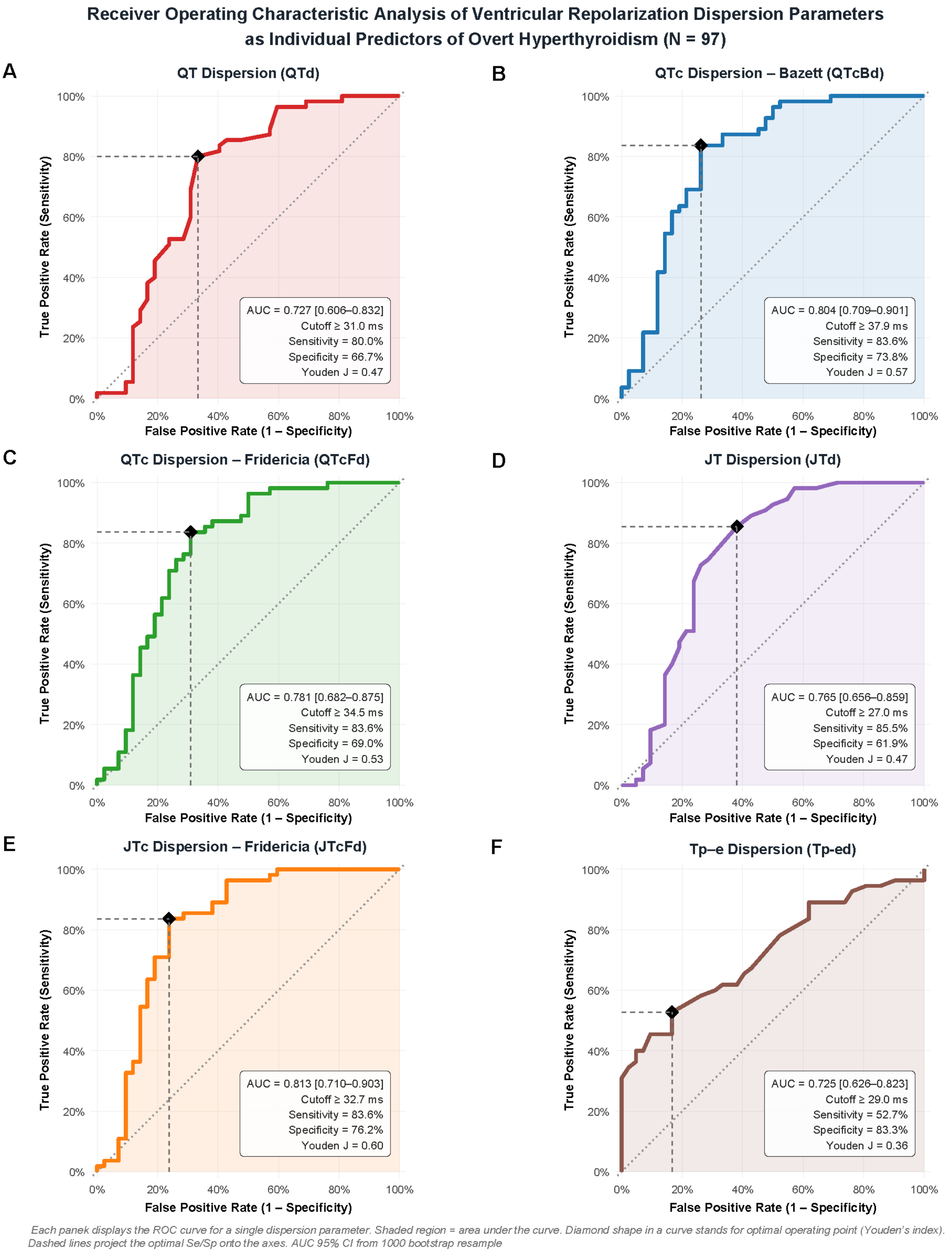

Figure S.6. *Individual Receiver Operating Characteristic Curves for Six Dispersion Parameters with Shaded AUC, Optimal Operating Points (Youden's Index), and Projected Sensitivity/Specificity: JTcFd (AUC = 0.813) and QTcBd (AUC = 0.804) Demonstrate the Highest Discriminatory Capacity*

Note. Each panel (A–F) displays the ROC curve for a single dispersion parameter. Shaded region = area under the curve. Diamond marker = optimal operating point (Youden's index). Dashed lines project the optimal sensitivity and specificity onto the respective axes. AUC with 95% bootstrap CI (1,000 resamples) and full diagnostic metrics (cutoff, sensitivity, specificity, Youden J) annotated within each panel.

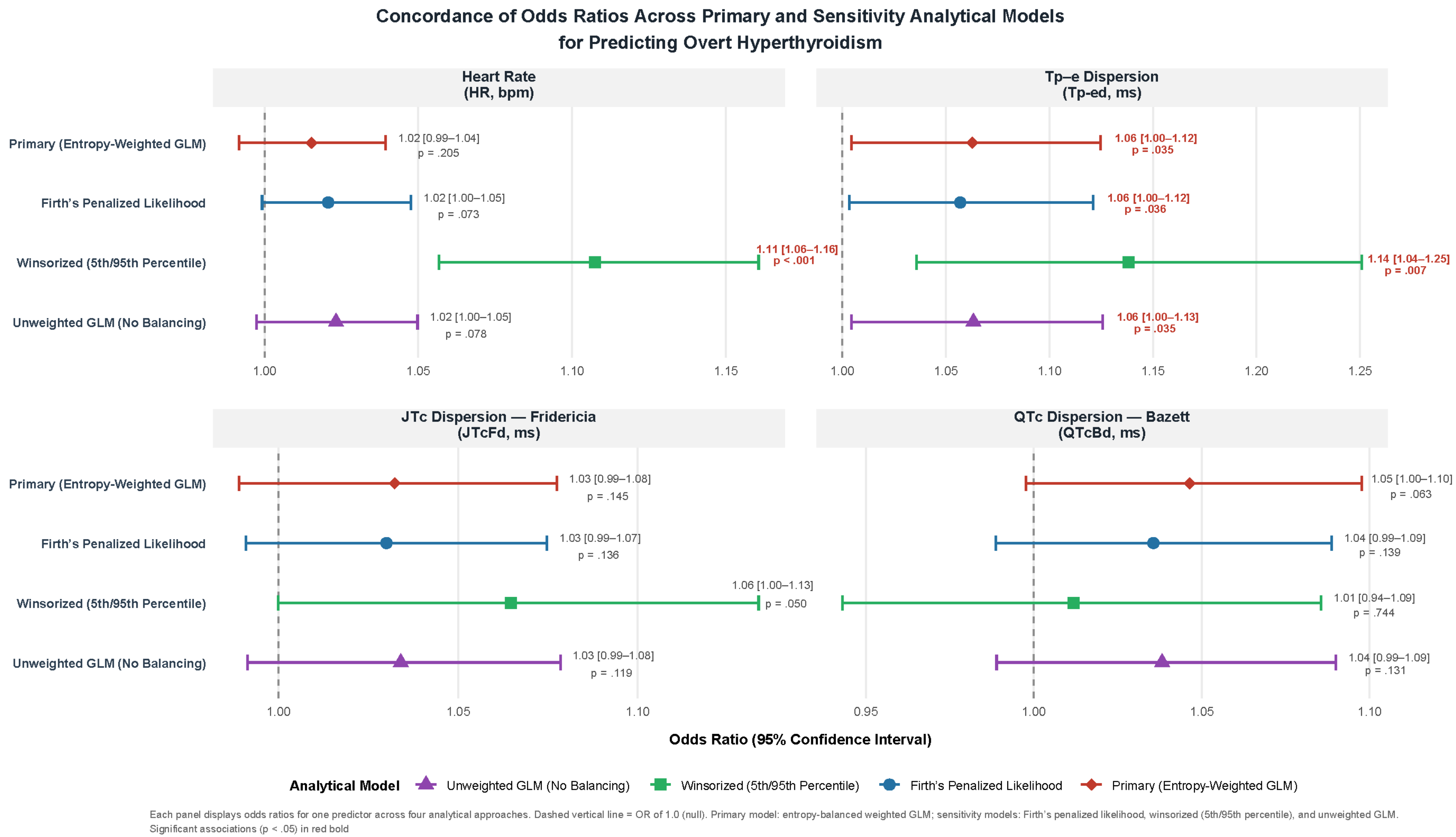

Figure S.7. *Forest Plot Comparing Odds Ratios Across Primary (Entropy-Weighted), Firth's Penalised, Winsorised, and Unweighted Models: Directional Concordance Across All Four Analytical Approaches for Each Predictor*

Note. Each panel corresponds to one predictor. Point estimates with 95% CIs are displayed for four analytical approaches: primary (entropy-weighted GLM, red diamond), Firth's penalised likelihood (blue circle), winsorised at 5th/95th percentiles (green square), and unweighted GLM (purple triangle). OR [95% CI] and p-values annotated beside each estimate. Dashed vertical line = OR of 1.0 (null hypothesis). Significant associations ($p < .05$) highlighted in red.

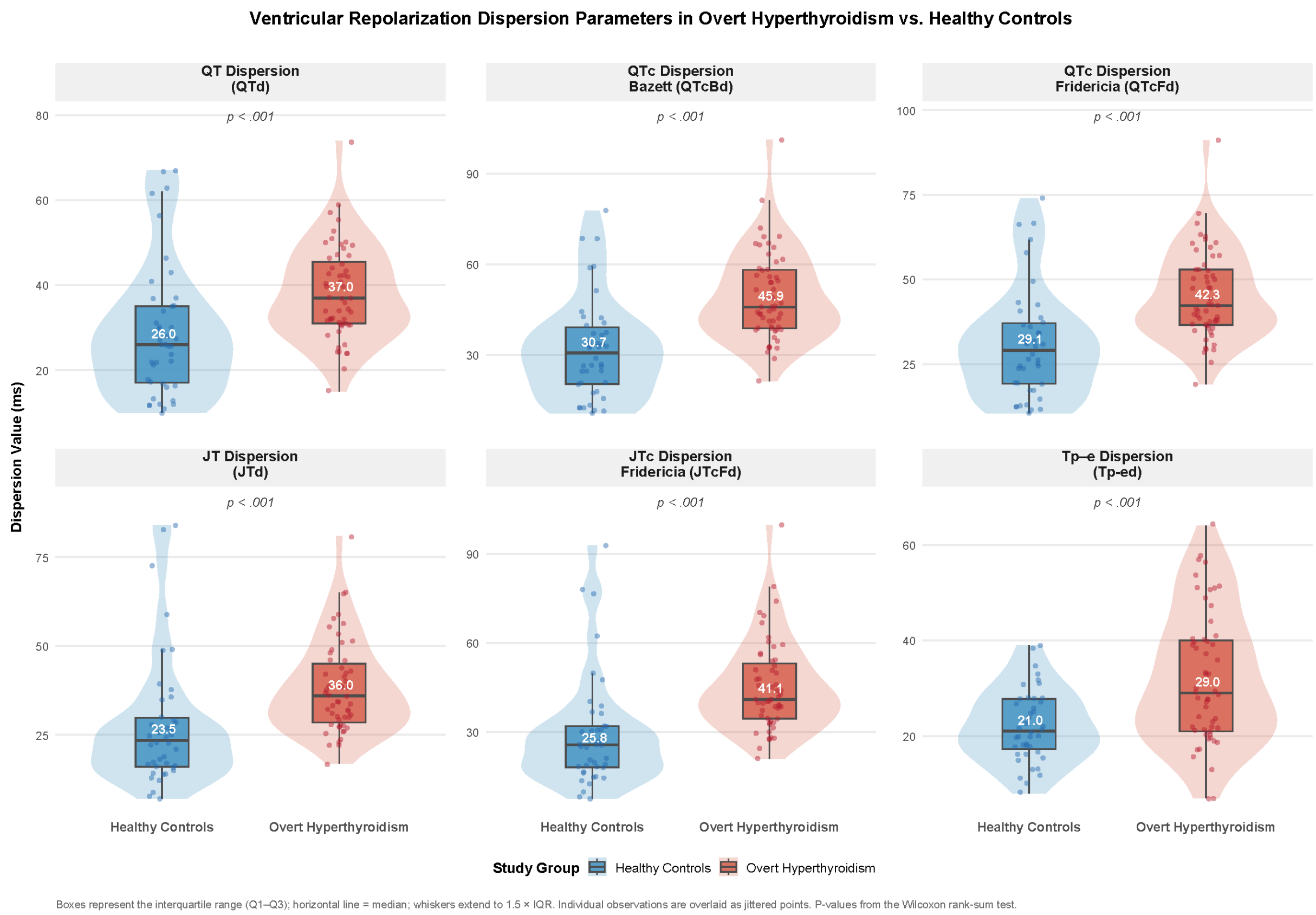

Figure S.8. *Violin-Box-Jitter Plots of Six Ventricular Repolarization Dispersion Parameters Stratified by Study Group: All Parameters Significantly Elevated in Overt Hyperthyroidism (All p < .001), with Individual Data Points Revealing Distributional Overlap*

Note. Semi-transparent violins depict distributional shape; boxes represent the interquartile range (Q1–Q3) with the horizontal line at the median; whiskers extend to 1.5 × IQR. Individual observations are overlaid as jittered points. Median values annotated in bold above each box. P-values from the Wilcoxon rank-sum test. Panels ordered by parameter family: QT-derived, JT-derived, Tp-e.

Figure S.9

Cook's Distance Influence Diagnostics for the Final Logistic Regression Model: No Single Observation Exceeds the 4/N Threshold, Confirming That Model Estimates Are Not Disproportionately Driven by Any Individual Case

Note. Each vertical segment represents one observation's Cook's distance. Red dashed horizontal line = conventional threshold at 4/N. Observations exceeding this threshold (if any) are highlighted in red. N = 97.

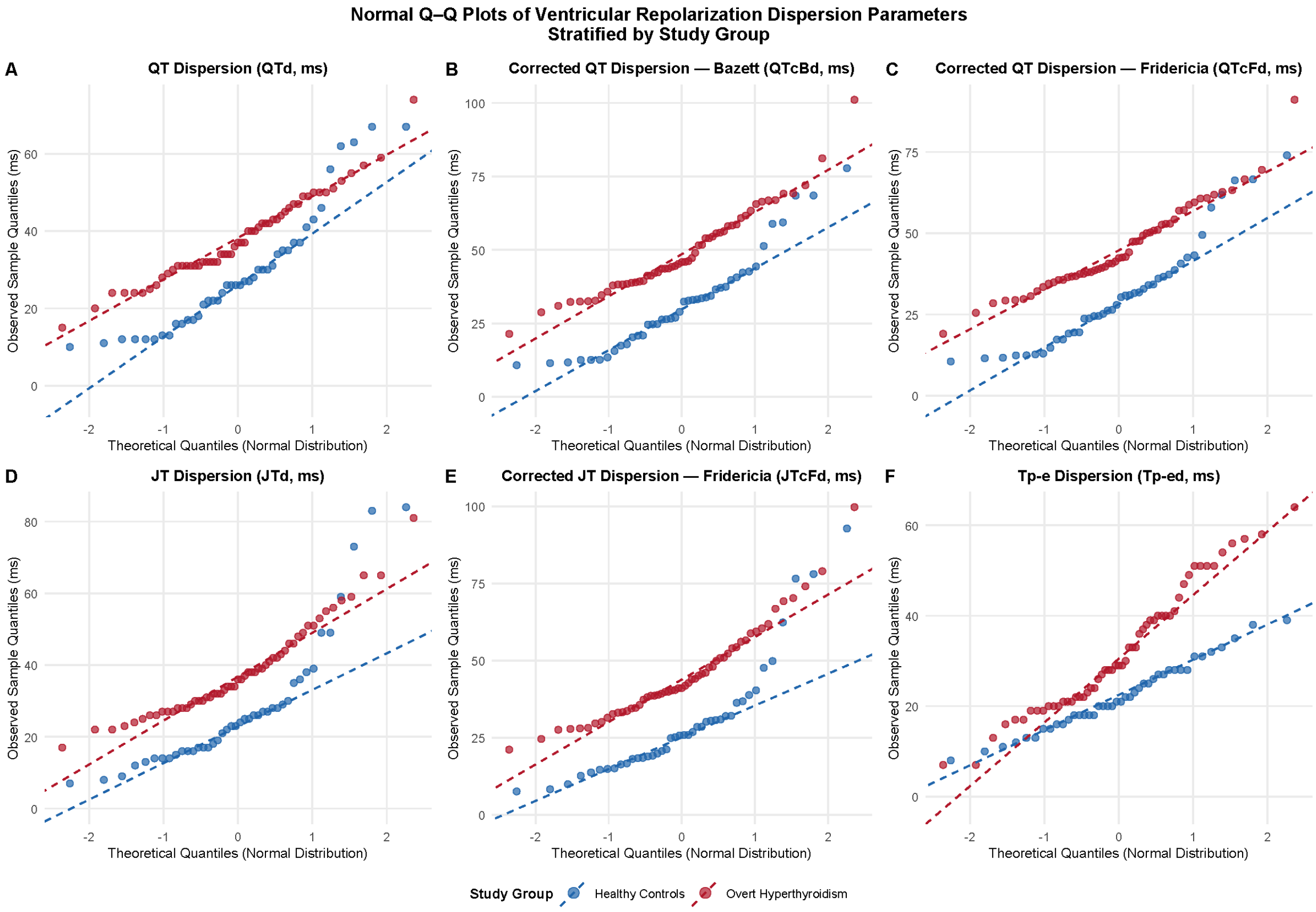

Figure QA-1. *Normal Q–Q Plots of Six Ventricular Repolarization Dispersion Parameters Stratified by Study Group: Systematic Deviation from the Theoretical Normal Line Corroborates the Non-Parametric Analytical Strategy*

Note. Panels A–F display quantile–quantile plots for each dispersion parameter, with observed sample quantiles (ms) plotted against theoretical normal quantiles. Dashed lines = expected trajectory under normality. Points colour-coded by group: blue = Healthy Controls, red = Overt Hyperthyroidism.

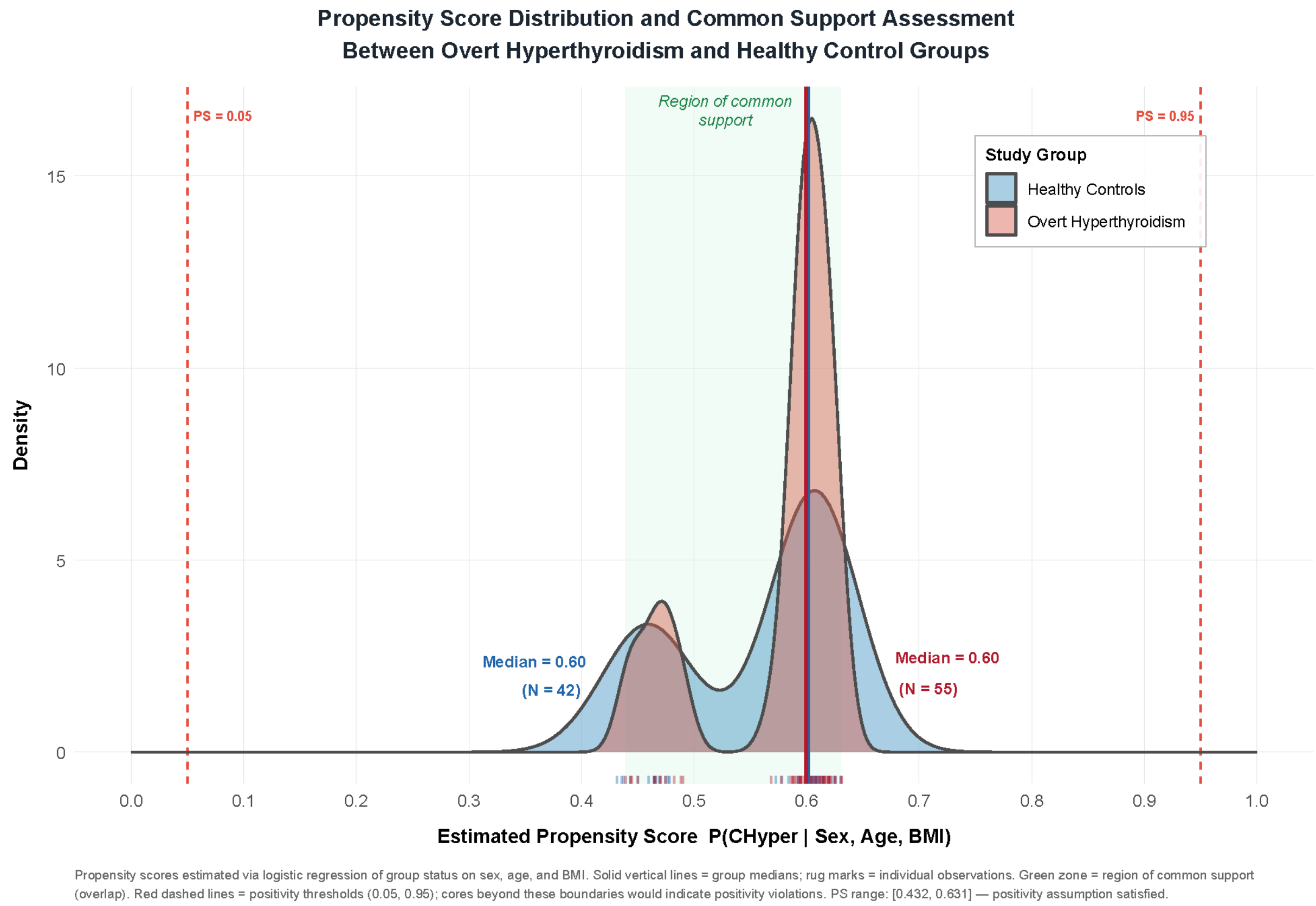

Figure QA-2. *Propensity Score Distributions and Common Support Assessment Between Overt Hyperthyroidism and Healthy Control Groups: Complete Overlap (PS Range 0.43–0.63) with No Positivity Violations Confirms the Validity of Entropy Balancing*

Note. Density curves display the distribution of estimated propensity scores [P(OHT | Sex, Age, BMI)] for each group. Solid vertical lines = group medians; rug marks along the x-axis = individual observations. Green-shaded zone = region of common support (overlap). Red dashed lines = positivity thresholds at PS = 0.05 and PS = 0.95; scores beyond these boundaries would indicate positivity violations.
